# Interprofessional Education Experience, Self-Rated Knowledge, and Perceived Interprofessional Collaboration Among Health Professions Faculty and Educators: A Cross-Sectional Mixed-Methods Study in Albania and Italy

**DOI:** 10.3390/healthcare14162649

**Published:** 2026-08-20

**Authors:** Viktorija Xharra, Rosario Caruso, Florian Spada, Ippolito Notarnicola, Sara Carrodano, Alessandro Stievano

**Affiliations:** 1 Department of Biomedicine and Prevention, University of Rome Tor Vergata, 00133 Rome, Italy; xharraviktorija@gmail.com; 2 Department of Biomedical Sciences for Health, University of Milan, 20133 Milan, Italy; 3 Health Professions Research and Evidence Transfer Unit, IRCCS MultiMedica, 20099 Sesto San Giovanni, Italy; 4 Department of Biomedical Sciences, Faculty of Medicine, University “Our Lady of the Good Counsel”, 1000 Tirana, Albania; f.spada@unizkm.al; 5 Department of Medicine and Surgery, University of “Kore”, Piazza dell’Università, 94100 Enna, Italy; ippolito.notarnicola@unikore.it; 6 Clinical Governance and Risk Management Department, ATS ASL5, 19124 La Spezia, Italy; carrodano.sara@gmail.com; 7 Department of Clinical and Experimental Medicine, University of Messina, 98100 Messina, Italy; alessandro.stievano@gmail.com

**Keywords:** interprofessional education, interprofessional collaboration, health professions education, faculty development, collaborative practice, mixed-methods research, teamwork

## Abstract

**Highlights:**

**What are the main findings?**

- After adjustment, self-reported prior IPE experience showed a clearer association with perceived collaboration than single-item self-rated knowledge; no interaction by country of work was supported.
- Perceived collaboration was highest for Cooperation and lowest for Partnership; participants’ written accounts provided complementary context by identifying professional silos, resistance to change, status hierarchies, and unclear role boundaries.

**What are the implications of the main findings?**

- In this sample, self-reported prior IPE experience showed a clearer association with perceived collaboration than did a single-item self-rating of IPE knowledge; this comparison should not be interpreted as showing that experience is inherently more important than knowledge.
- Curricular revision and shared teaching (33.3%) was the most frequently proposed strategy, but the barriers described were predominantly cultural, organisational and resource-related, indicating that curricular change alone is unlikely to be sufficient without institutional and faculty-level support.

**Abstract:**

**Background/Objectives**: Interprofessional education (IPE) supports collaborative practice, but educational exposure, self-rated knowledge, and collaboration are distinct constructs. We examined the adjusted associations between self-reported prior IPE experience and single-item self-rated IPE knowledge and perceived collaboration among health professions faculty and educators in Albanian and Italian work contexts. **Methods**: This quantitative-dominant cross-sectional mixed-methods study included 445 faculty members and educators. Collaboration was assessed using language-matched versions of the 23-item Assessment of Interprofessional Team Collaboration Scale II (AITCS-II). Self-rated IPE knowledge was assessed using a single author-developed 0–100 item. Heteroskedasticity-consistent type 3 standard errors in linear regressions examined adjusted associations and exploratory context interactions. Confirmatory factor analysis and reliability analyses examined preliminary evidence of internal structural validity and internal consistency in this sample. Directed qualitative content analysis with inductive expansion examined 341 responses from 114 participants and was integrated into the aggregate results. **Results**: A total of 47.2% of participants reported prior IPE experience; the mean single-item self-rated IPE knowledge score was 31.7/100, and the mean overall AITCS-II score was 3.95/5. Cooperation was rated highest and Partnership lowest. In the adjusted model (n = 417), self-reported prior IPE experience was associated with a 0.32-point higher overall AITCS-II score (95% CI 0.18–0.47), while each 10-point increase in the single-item knowledge rating was associated with a 0.03-point higher score (95% CI 0.002–0.058). No country-of-work interaction was statistically supported. The response covariance structure was compatible with the hypothesised three-factor model. Qualitative findings emphasised experiential and longitudinal learning and identified professional silos, hierarchies, curricular rigidity, limited resources, and weak organisational coordination as barriers. **Conclusions**: Self-reported prior IPE experience showed a clearer and more consistent association with perceived collaboration than did the single-item measure of self-rated IPE knowledge, whose association was small and less consistent across domains. Applied, repeated, and contextually embedded IPE, supported by faculty development and organisational structures, may facilitate collaborative practice. Causal and cross-country conclusions are not warranted.

## 1. Introduction

Contemporary healthcare increasingly relies on professionals from different disciplines working together to address complex and interdependent patient needs. Interprofessional collaboration encompasses more than the parallel contribution of different professions: it requires communication, clarity, role recognition, coordinated action, shared responsibility, and participation in decision-making. These processes operate at individual, team, and organisational levels and can influence professionals, healthcare organisations, and patients [1,2]. Nevertheless, evidence linking interprofessional collaboration directly to patient-reported outcomes remains heterogeneous and methodologically limited, which calls for careful distinction between perceived collaboration, collaborative behaviour, and downstream patient outcomes [3].

Interprofessional education (IPE) is intended to prepare health professionals for collaborative practice by enabling members of two or more professions to learn about, from, and with one another [4]. Current competency frameworks emphasise communication, roles and responsibilities, values and ethics, teamwork, and person-centred care [5,6]. Recent evidence indicates that IPE can improve interprofessional knowledge, attitudes, mutual respect, role clarity, communication, and teamwork-related competencies [6,7]. Educational exposure, knowledge about IPE, and collaboration in practice are nevertheless related but non-interchangeable constructs.

This distinction is important because much of the IPE literature assesses proximal educational outcomes rather than transfer to professional behaviour. Evaluations commonly report favourable reactions, knowledge, attitudes, or self-perceived competencies, whereas comparatively few assess behavioural change or patient- and system-level outcomes [8,9]. Self-report instruments may also capture perceived preparedness rather than observable collaborative behaviour [10]. Knowledge of IPE therefore does not necessarily ensure its enactment in collaborative practice. Translation into practice may depend on the quality and continuity of prior IPE experience: authentic scenarios, experiential learning, repeated exposure, and skilled facilitation may support application, whereas brief or isolated activities may not produce sustained change [9,11]. Moreover, IPE interventions may rely on an undifferentiated concept of teamwork and fail to distinguish operational cooperation from more demanding forms of partnership involving reciprocal recognition, power-sharing, and collective decision-making [12]. Professionals may therefore coordinate tasks and cooperate without experiencing equivalent participation in decisions or shared responsibility.

The implementation of IPE is context-dependent. Influences operate across micro-level teaching conditions, meso-level institutional structures, and macro-level educational and sociocultural systems, with leadership, resources, faculty development, administrative coordination, and sustainability as recurring determinants [13]. Faculty members shape curricula and model professional relationships, but inadequate resources, insufficient preparation, curricular fragmentation, professional boundaries, and weak institutional support may constrain implementation [14,15]. Hierarchies, conflicting professional norms, and unfamiliarity among team members may likewise impede collaboration even when its value is recognised [16]. Because these determinants are context-sensitive, the relationships between IPE experience, knowledge, and perceived collaboration may not be uniform across working settings. Country of work may capture part of this variation, although it remains only a partial proxy for institutional, educational, and sociocultural conditions.

Evidence on IPE in Albania has begun to accumulate but has mainly focused on learners. Readiness for interprofessional learning among undergraduate healthcare students in Albania has been examined through the cross-cultural adaptation of the Readiness for Interprofessional Learning Scale [17], a single-centre cross-sectional study [18], and a clustering analysis identifying heterogeneous readiness profiles across health professions programmes [19]. Collectively, this work suggests generally favourable dispositions towards interprofessional learning, alongside variability across learner profiles and programmes. Readiness to learn interprofessionally, however, is an antecedent of educational participation rather than evidence that collaboration is subsequently experienced within professional working relationships.

Faculty have been studied primarily in terms of attitudes and readiness towards IPE [14,15], rather than the collaboration they themselves experience within their working relationships. This distinction is relevant because faculty and educators play a central role in determining whether interprofessional content is embedded in curricula, allocating the time and settings in which it occurs, and modelling the professional relationships observed by students. Their prior IPE experience, knowledge of IPE, and perceptions of collaboration may therefore be relevant to whether IPE is implemented and to the form it takes [7,17,18,19]. Examining these dimensions in Albanian and Italian contexts offers an opportunity to assess whether their relationships with perceived collaboration are broadly consistent or contextually contingent.

The 23-item Assessment of Interprofessional Team Collaboration Scale II (AITCS-II) conceptualises perceived collaboration across Partnership, Cooperation, and Coordination and was developed for practitioners working in healthcare teams [20,21]. Its Italian version, which is I-AITCS-II, retained the hypothesised three-domain structure, which explained 51% of the total variance, and showed acceptable model fit (RMSEA = 0.054, 95% CI 0.039–0.088; CFI = 0.960; TLI = 0.948; SRMR = 0.076). Internal consistency was high for the overall scale (Cronbach’s α = 0.968) and its domains (α = 0.923–0.944), while the scale-level content validity index was 0.98 [22]. Examining these related but distinct domains may clarify whether respondents primarily perceive operational cooperation or also experience reciprocal participation and shared responsibility. Among faculty and educators, however, interprofessional working relationships may span educational, organisational, and clinical settings rather than a single, clearly bounded healthcare team.

Previous research has associated IPE experience with more favourable perceptions of interprofessional teamwork among health professions students [23], while perceptions of collaboration have also been shown to vary across professional groups and working environments [24]. It remains unclear whether prior IPE experience and self-rated IPE knowledge are each associated with perceived collaboration among faculty and educators after mutual adjustment and adjustment for measured covariates, and whether these relationships vary by country of work. Quantitative associations alone cannot establish which forms of experience were considered meaningful, why knowledge may or may not be translated into practice, or which educational, organisational, and relational conditions remain outside the statistical model. Written accounts of strategies for strengthening IPE, perceived implementation barriers, and the importance attributed to IPE may help explain and extend these associations by identifying conditions that may facilitate or constrain the translation of educational exposure into collaborative practice.

For these reasons, this quantitative-dominant mixed-methods study primarily aimed to examine the adjusted associations of self-reported prior IPE experience and self-rated IPE knowledge with perceived interprofessional collaboration among health professions faculty and educators working in Albanian and Italian contexts. Secondary aims were to describe self-reported prior IPE experience, self-rated IPE knowledge, and AITCS II domain scores and to examine preliminary evidence of internal structural validity and internal consistency for the AITCS-II scores in the study sample. The study also explored whether these associations varied by country of work. Finally, in a nested subsample of respondents who provided analysable written responses, a directed qualitative content analysis informed by the quantitative findings and allowing inductive expansion was used to contextualise and extend selected results by identifying educational, organisational, and relational conditions that may facilitate or constrain the translation of IPE into collaborative practice.

## 2. Materials and Methods

### 2.1. Study Design, Reporting Framework, and Analytical Questions

This observational cross-sectional study used a quantitative-dominant mixed-methods design with an embedded qualitative component [25]. Quantitative measures and open-ended responses were collected concurrently within the same survey. Quantitative analyses were completed first and informed the subsequent directed qualitative content analysis. The analytical sequence therefore did not represent a temporal sequence of data collection. The qualitative component involved a nested subsample of respondents who provided analysable written responses and was used to contextualise and extend selected quantitative findings. The geographical composition of this subsample did not mirror that of the overall quantitative sample; consequently, no representative qualitative comparison between the Albanian and Italian working contexts was planned.

No single reporting guideline fully addresses all components of this mixed-methods design. Reporting was therefore guided by the Strengthening the Reporting of Observational Studies in Epidemiology (STROBE) statement for the cross-sectional quantitative strand [26], the Standards for Reporting Qualitative Research (SRQR) for the qualitative component [27], and the Good Reporting of A Mixed Methods Study (GRAMMS) criteria for the overall design and integration [28]. Completed STROBE, SRQR, and GRAMMS reporting checklists are provided as Supplementary Files S2–S4, respectively.

The primary quantitative analysis examined the associations of self-reported prior IPE experience and self-rated IPE knowledge with the AITCS-II overall score after mutual adjustment and adjustment for the prespecified measured covariates [29]. Effect modification by country of work was examined as an exploratory extension. An ancillary analysis examined the expected concordance between self-reported prior IPE experience and self-rated IPE knowledge. Because both indicators were self-reported and partly conceptually overlapping, this analysis was not used to assess the validity of the knowledge item or to infer that self-reported prior IPE experience produced greater knowledge.

### 2.2. Study Setting, Participants, Sample Size, and Recruitment

The study involved faculty members and educators working in health professions education in Albanian and Italian contexts. Three universities participated in Albania: one delivered health professions programmes in Italian and two delivered programmes in English. In Italy, participants were recruited through three universities: one located in Northern Italy, one in Central Italy, and one in Southern Italy. Data were collected from 7 May to 15 July 2026.

Eligible participants were affiliated with one of the participating universities and, during the data-collection period, were directly involved in teaching, tutoring, mentoring, supervising, or coordinating the education of students enrolled in health professions programmes. Eligible roles included faculty members, researchers with teaching or supervisory responsibilities, teaching assistants, tutors, and clinical or academic mentors. Administrative or technical staff without direct educational responsibilities, researchers without a teaching or supervisory role, individuals not involved in health professions education, and personnel on extended leave throughout the data-collection period were excluded.

An a priori sample size calculation was conducted in G*Power 3.1 using the fixed-model multiple regression procedure for testing an increase in (R^2^) [30]. The calculation specified two tested predictors and nine total predictors in the full model. Assuming a small but potentially relevant incremental effect size of (f^2^ = 0.03), a two-sided alpha level of 0.05, and 80% statistical power, 325 evaluable participants were required. The recruitment target was increased to 350 participants to allow for approximately 7% analysis-specific data loss due to incomplete outcome or covariate information [31].

Assuming that approximately 70% of invited individuals would provide an eligible and analytically usable questionnaire, 500 faculty members and educators were invited: 400 through the participating Italian universities and 100 through the participating Albanian universities. No within-list subsampling or random selection was performed. The invitation stage therefore represented a complete enumeration of the accessible institutional lists, although these lists constituted a convenience-based institutional sampling frame rather than a population register of all health professions faculty and educators in the two countries. The allocation reflected the relative sizes of the accessible lists and was not intended to produce equally sized working-country groups. The planned sample size was intended to support the pooled primary analysis; country-of-work interaction analyses were exploratory and were not separately powered to demonstrate contextual differences.

The principal investigator distributed the initial invitations through institutional communication channels. Each invitation included a link to a Research Electronic Data Capture (REDCap) form containing information about the study, eligibility criteria, and the informed-consent statement [32]. The invitation also indicated that participants could request a paper questionnaire. Up to three reminders were sent at approximately 10-day intervals over the following month. Local investigators and academic or programme coordinators additionally encouraged eligible faculty members and educators to participate through personal contacts. Participants confirmed their eligibility and provided informed consent before completing either the electronic or paper questionnaire. Completed paper questionnaires were subsequently transcribed faithfully into the corresponding REDCap fields by a member of the research team (V.X.). No information was collected on the characteristics of, or reasons for non-participation among, individuals who did not return a questionnaire.

The context variable denoted the participant’s current country of work rather than nationality, citizenship, or the country through which the invitation was distributed. Responses explicitly identifying Albania were assigned to the Albanian working context. In Italy, two separate REDCap questionnaire containers were used for operational purposes and to distinguish recruitment channels: the container labelled “Italia” collected responses through the Northern Italian recruitment stream, whereas the container labelled “Italy2” collected responses through the Central and Southern Italian recruitment streams. Responses from both containers were combined into a single Italian country-of-work category for the primary analyses. Free-text and “Other” responses were manually assigned to Albania or Italy only when the available information supported the classification, and all mapping decisions were retained in an auditable decision file. Records for which country of work could not be resolved were retained in analyses that did not require this variable and excluded from country-stratified and interaction analyses.

Survey source identified the technical REDCap container and associated recruitment channel rather than an independent contextual exposure. It was retained as a data-provenance variable and was not treated as interchangeable with country of work. The coding and analytical roles of country of work and survey source are described further in Section 2.3.4.

All consenting records with a non-missing technical REDCap record identifier were eligible for the descriptive analyses. The identifier was generated automatically for data-management purposes and could not be used by the analytical team to identify or contact individual participants. No directly identifying information was included in the analytical export. Analysis-specific samples were defined according to the availability of the variables required for each analysis. Participant flow from the full sample to the psychometric, regression, and nested qualitative samples is reported in Supplementary Figure S1.

### 2.3. Data Collection and Measures

#### 2.3.1. Survey Administration and Structure

Data were collected using a web-based self-report questionnaire implemented in REDCap [32]. Faculty members who preferred to complete a paper version could request it by responding to the study invitation. The completed paper questionnaires were subsequently entered into REDCap by a member of the research team (V.X.), with responses transcribed faithfully into the corresponding electronic fields. No directly identifying information was requested, and analyses were conducted using a de-identified data export. The questionnaire included participant and professional characteristics, self-reported prior IPE experience, self-rated IPE knowledge, the AITCS-II, and three optional open-ended questions. Quantitative measures and open-ended responses were collected during the same survey administration.

The questionnaire was available in Italian and English, and respondents selected their preferred language. The Italian version included the I-AITCS II, previously evaluated for validity and reliability among healthcare providers [22], whereas the English version included the revised English AITCS-II [21]. Before pooling the data, the two language versions were verified as having equivalent item content and order, response anchors, scoring direction, and domain allocation.

When completing the AITCS-II, participants were presented with the introductory stem, i.e., “When we are working as a team, all of my team members…”. The questionnaire defined a team as regular interactions between one or more health professionals for the purpose of providing patient care. The intended frame of reference was therefore interprofessional teamwork related to patient care. However, participants were not asked to identify a specific team, clinical service, or working environment, or to indicate whether their responses drew on concurrent clinical practice, their working relationships as educators, or both. Because faculty members and educators may hold overlapping academic, organisational, and clinical roles, the frame of reference actually applied by each respondent could not be verified. AITCS-II scores were therefore interpreted as perceived interprofessional collaboration with an intended healthcare-team referent, subject to possible between-participant heterogeneity in the professional relationships considered.

#### 2.3.2. Self-Reported Prior IPE Experience and Self-Rated IPE Knowledge

Self-reported prior IPE experience was elicited using the question, “Have you had direct experience in Interprofessional Education activities?”, with binary response options (yes/no). No operational definition or examples of qualifying activities were provided. The item did not specify whether the experience had occurred as a student, faculty member, facilitator, or healthcare professional, or whether it involved formal or informal activities. It was therefore interpreted as a broad, respondent-defined indicator of previous exposure to activities perceived as IPE, rather than as documentation of a defined educational intervention.

Single-item self-rated IPE knowledge was elicited using the question, “How well do you think you know the methods and approaches of Interprofessional Education?” Responses were recorded on a visual analogue scale coded from 0 to 100, with the verbal anchors “Not at all”, “Fairly well”, and “Completely” displayed at the left, centre, and right of the scale, respectively. Higher values indicated greater self-perceived familiarity with and understanding of IPE [33]. These properties were not specifically evaluated for the present author-developed item; it was therefore interpreted as an approximate self-rating rather than as a comprehensive or psychometrically validated measure of IPE knowledge.

It was not designed to assess factual knowledge or mastery of a predefined IPE knowledge domain and was not treated as a comprehensive or psychometrically validated measure of IPE knowledge. Prior experience and self-rated knowledge were therefore retained as distinct author-developed indicators: the former represented reported exposure to IPE, whereas the latter represented respondents’ subjective appraisal of their current understanding. Because knowledge ratings showed a substantial concentration at zero and frequent heaping at multiples of 5 or 10, these distributional features were addressed explicitly in an ancillary concordance analysis.

#### 2.3.3. Perceived Interprofessional Collaboration

Perceived interprofessional collaboration was assessed using language-matched versions of the Assessment of Interprofessional Team Collaboration Scale II: the Italian I-AITCS II, previously evaluated for validity and reliability among healthcare providers [22], and the revised English AITCS-II [21]. The instrument comprises 23 items rated from 1 (never) to 5 (always) and includes three related domains: Partnership (8 items), Cooperation (8 items), and Coordination (7 items). Domain and overall scores were calculated as the mean of the available constituent items, with higher values indicating stronger perceived collaboration.

Scores were calculated when at least 80% of the relevant items had been completed: at least 7 of 8 Partnership items, 7 of 8 Cooperation items, 6 of 7 Coordination items, and 19 of all 23 items for the overall score. No missing individual item was imputed.

The continuous overall AITCS-II mean score was the primary outcome because the study examined whether prior IPE exposure and self-rated knowledge were associated with perceived interprofessional collaboration. Complete-item scoring was examined as a sensitivity analysis. No dichotomous threshold was used to classify collaboration because no validated cut-off for the AITCS-II mean score was available.

#### 2.3.4. Covariates and Context Variables

The theory-informed adjustment set comprised country of work, profession, years of work experience, annual teaching hours, and academic role. Profession was grouped as physician, nurse, another specified health profession (physiotherapist, midwife, or psychologist), or other/unspecified profession.

Years of work experience were modelled per 10-year increase. Annual teaching hours showed a right-skewed distribution and were entered as log(1 + x) in the final analytical dataset [34]. Academic role was classified as junior or senior according to the response categories used in the questionnaire. Age was described but excluded from the primary adjustment set because of its strong conceptual and empirical overlap with years of work experience. The adjustment set was specified a priori from variables measured consistently across the survey sources and considered plausibly related to both prior IPE exposure and perceived collaboration. Detailed information on institutional characteristics, previous formal teamwork training, clinical versus predominantly academic employment, leadership responsibilities, type of programme taught, and current participation in interprofessional teams was not available in sufficient detail and could therefore not be included in the adjustment set.

Survey source identified the technical REDCap container and associated recruitment channel. It was excluded from the primary models because it was partially confounded with country of work and, within Italy, with geographical recruitment area. In particular, the second technical container included only participants recruited in Central and Southern Italy. Survey source was therefore added only in a sensitivity analysis to quantify the resulting changes in estimates and precision. Complete response formats, scoring rules, covariate coding, and analytical roles are provided in Supplementary Table S1.

#### 2.3.5. Open-Ended Questions

The optional open-ended section elicited written responses concerning strategies for strengthening IPE (“How could IPE be integrated into or strengthened within the academic context?”), perceived barriers to its implementation (“What obstacles or difficulties do you perceive in promoting IPE?”), and the importance attributed to IPE (“How important do you consider IPE to be for students’ education?”). The wording of the questions in both survey languages is provided in Supplementary Table S1. Responses were allowed in Italian, Albanian, or English.

The third question primarily elicited an intensity judgement or evaluative position through a free-text response. Its analysis therefore distinguished between the level of importance expressed and any accompanying explanatory rationale.

### 2.4. Quantitative Analysis

Analyses were conducted using R version 4.6.1 (R Foundation for Statistical Computing, Vienna, Austria). All tests were two-sided, 95% confidence intervals were reported, and an alpha level of 0.05 was used, with multiplicity adjustment where specified.

#### 2.4.1. Data Preparation, Scoring, and Missing Data

Data-quality checks addressed duplicate technical identifiers, out-of-range responses, inconsistencies between recorded and recalculated scale scores, the number of completed AITCS-II items, and missingness across all analytical variables. Country-of-work responses requiring manual classification were mapped using a separate decision file, and all classification decisions were documented. No out-of-range values were retained.

Available-item scoring was applied according to the documented minimum-item requirements, without imputation of individual missing items. Confirmatory factor analysis and regression models used analysis-specific approaches to missing data, as described below. Regression analyses were conducted using complete cases for the variables included in each model. Data-quality findings and variable-level missingness are reported in Supplementary Table S2, and the missing-data profile is shown in Supplementary Figure S2. The de-identified dataset is published elsewhere [35].

#### 2.4.2. Descriptive Analysis

Categorical variables were summarised using frequencies and percentages. Continuous variables were summarised using means and standard deviations or medians and interquartile ranges, according to their observed distributions. Self-reported prior IPE experience, self-rated IPE knowledge, and AITCS-II domain and overall scores were described for the full sample and by country of work. Country-stratified summaries were interpreted descriptively and were not used to establish national differences.

Item-level response distributions, missingness, floor effects, and ceiling effects were examined for all 23 AITCS-II items. The proportion of respondents selecting the highest response category was summarised by item and domain because upper-end response concentration could reduce discrimination among participants reporting relatively high perceived collaboration. Item-level distributions and corrected item–total correlations are reported in Supplementary Table S3.

#### 2.4.3. Preliminary Examination of Internal Structural Validity and Internal Consistency

Internal consistency was examined using Cronbach’s alpha, standardised alpha, ordinal alpha based on polychoric correlations, mean inter-item correlations, and composite reliability for each confirmatory factor [36]. Composite reliability was calculated from the standardised factor loadings and indicator residual variances. Very high reliability coefficients were interpreted alongside mean inter-item correlations as possible evidence of item redundancy rather than as an unqualified indication of measurement quality.

The hypothesised correlated three-factor structure was examined using confirmatory factor analysis. Items were treated as ordered categorical variables, and the model was estimated using weighted least squares with mean and variance adjustment, theta parameterisation, and pairwise handling of missing item responses. Model fit was evaluated using the comparative fit index, Tucker–Lewis index, root mean square error of approximation with its 90% confidence interval, and standardised root mean square residual. Exact fit indices were considered jointly rather than relying on any single criterion. Standardised factor loadings, residual correlations, and modification indices were inspected for diagnostic purposes. No residual correlations were added, and no data-driven model respecification was undertaken. These analyses examined whether the covariance structure of the item responses was compatible with the hypothesised three-factor model and whether the resulting scores showed adequate internal consistency. They were interpreted only as providing preliminary evidence of internal structural validity and internal consistency in this sample. They did not evaluate the content validity of the AITCS-II for educational or organisational working relationships, validate the instrument for faculty or educator populations, or establish whether the construct had the same meaning as in clinical-team populations.

Multigroup ordinal measurement invariance across countries of work was not estimated because several country-by-item response categories were sparse or empty. As a pragmatic sensitivity analysis, the items were treated as continuous, and multigroup models were estimated using robust maximum likelihood with full-information maximum likelihood for missing data.

Configural, metric, and scalar models were compared; strict invariance was not tested because it could not be established due to sparse response categories. Changes in fit between successive models were described using ∆CFI and ∆RMSEA. Absolute changes not exceeding 0.010 for CFI and 0.015 for RMSEA were treated as pragmatic support for invariance [37]. These criteria were applied descriptively because the ordinal items were treated as continuous and the country groups were unequal. The analysis was therefore interpreted only as supportive sensitivity evidence and not as establishing ordinal measurement invariance. Reliability, factor analysis, diagnostic, and invariance-sensitivity results are reported in Supplementary Table S4a–f and Supplementary Figure S3.

#### 2.4.4. Ancillary Concordance Analysis

The expected concordance between self-reported prior IPE experience and single-item self-rated IPE knowledge was examined as an ancillary analysis. Because knowledge scores showed a marked concentration at zero, a two-part model was used. First, logistic regression estimated the adjusted odds of reporting any self-rated IPE knowledge, defined as a score greater than zero. Second, among respondents reporting positive knowledge values, ordinary least squares regression with heteroskedasticity-consistent HC3 standard errors estimated the adjusted mean difference in self-rated knowledge. Both components were adjusted for the same prespecified covariates included in the primary analysis.

Median regression was retained as a supplementary sensitivity analysis because of the heaping and non-normal distribution of the 0–100 knowledge scores. The concentration at zero and frequent use of multiples of 5 or 10 suggested that respondents may have used the scale as an approximate judgement of familiarity or confidence rather than as a finely graded continuous estimate. These analyses were used only to describe expected concordance between two related self-reported indicators. They were not interpreted as evidence of construct or criterion validity, diagnostic accuracy, an educational effect, or a causal relationship between prior experience and knowledge.

Conventional linear and exploratory working-context interaction models for the experience–knowledge relationship were retained in Supplementary Table S6a–e for analytical transparency but were treated as ancillary sensitivity analyses because of the distributional characteristics and partly overlapping content of the two self-reported indicators.

#### 2.4.5. Primary Association Models and Exploratory Context Interactions

Bivariate monotonic relationships among self-rated IPE knowledge, AITCS-II domain and overall scores, and continuous participant characteristics were described using Spearman correlation coefficients. The complete correlation matrix is reported in Supplementary Table S5 and displayed as a heatmap in Supplementary Figure S4.

The primary outcome was the continuous overall AITCS-II mean score. Associations were estimated using ordinary least squares regression with heteroskedasticity-consistent HC3 standard errors and 95% confidence intervals. HC3 standard errors were used throughout to reduce reliance on the assumption of homoskedastic residual variance. Self-rated IPE knowledge was scaled per 10-point increase.

A fixed hierarchical model sequence was used. Two initial adjusted models examined self-reported prior IPE experience and self-rated knowledge separately, together with the theory-informed covariates. A joint adjusted model then included both focal predictors and all theory-informed covariates. This sequence examined whether each IPE-related indicator remained associated with perceived collaboration after mutual adjustment and adjustment for the prespecified measured covariates.

Effect modification by country of work was evaluated as an exploratory extension using a theory-guided sequence rather than automated stepwise selection. The first interaction model added the interaction between country of work and self-reported prior IPE experience to the joint adjusted model. The second interaction model added the interaction between country of work and self-rated knowledge while retaining the first interaction. Both interaction terms were retained in the final interaction model to estimate country-specific contrasts irrespective of their statistical significance.

Incremental (R^2^), adjusted (R^2^), Akaike information criterion, Bayesian information criterion, and HC3-robust tests of the interaction terms were reported. Country-specific estimates and 95% confidence intervals were derived from the fitted interaction model using appropriate linear combinations of the regression coefficients. Coefficients were interpreted as associations rather than causal effects. The interaction analyses were exploratory, were not separately powered for definitive subgroup comparisons, and were not used to assert national differences.

Collinearity was assessed using variance inflation factors. Model diagnostics included residual-versus-fitted and quantile–quantile plots, formal assessment of heteroskedasticity and functional form, and examination of Cook’s distances for potentially influential observations. Diagnostic findings were interpreted alongside the HC3-robust estimates; observations were not excluded solely on the basis of an influence statistic.

Full coefficients, collinearity diagnostics, model diagnostics, interaction tests, and country-specific estimates are reported in Supplementary Table S6a–e. Regression diagnostic plots are provided in Supplementary Figure S5.

#### 2.4.6. Sensitivity Analyses and Multiplicity

Sensitivity analyses included separate models for Partnership, Cooperation, and Coordination; complete-item AITCS-II scoring; inclusion of a quadratic term for self-rated knowledge; median regression for the knowledge outcome; and regression models additionally adjusted for survey source.

The Benjamini–Hochberg procedure was used to control the false discovery rate across corresponding coefficients in the three domain-specific models. Adjustment was applied separately to the self-reported prior IPE experience coefficients and the self-rated knowledge coefficients across Partnership, Cooperation, and Coordination. The primary overall-score analysis was not included in this multiplicity adjustment.

All sensitivity analyses, including the ancillary two-part concordance model and the source-adjusted estimates, are reported in Supplementary Table S7a–g.

### 2.5. Qualitative Component

#### 2.5.1. Qualitative Subsample and Corpus

The open-ended section was optional. The three open-ended questions were available across all questionnaire containers, and no survey source was excluded a priori. Completion was not required, and reasons for providing or not providing written responses were not collected. The qualitative component included participants who provided at least one valid respondent-generated response to one of the three open-ended questions. The final qualitative corpus comprised 114 of the 445 survey participants (25.6%) and included 341 written responses: 114 concerning strategies for strengthening IPE, 113 concerning perceived barriers to its implementation, and 114 concerning the importance attributed to IPE. No survey source or questionnaire container was excluded a priori. Consequently, the qualitative component represented a nested voluntary-response subsample and did not mirror the geographical or survey-source composition of the overall quantitative sample. Qualitative subsample characteristics are reported in Supplementary Table S8.

#### 2.5.2. Directed Qualitative Content Analysis

Written responses were analysed using directed qualitative content analysis with inductive expansion [38], informed by the analytical process described by Assarroudi et al. [39]. The primary level of abstraction was manifest content. Interpretations extending beyond the explicit textual meaning were recorded separately and required justification [40]. Responses were coded in their original language by one author proficient in Italian, Albanian, and English (V.X.). Albanian responses and excerpts selected for reporting in English were translated by V.X. and independently checked by a second author (A.S.). Translation discrepancies were resolved through discussion.

After completion of the quantitative analyses, an initial analytical matrix was developed using the Partnership, Cooperation, and Coordination domains of the AITCS-II and selected quantitative patterns selected for contextualisation or expansion. These patterns included the clearer and more consistent association observed for self-reported prior IPE experience, alongside the smaller and less precisely estimated association for self-rated IPE knowledge; relatively high perceived collaboration despite limited self-reported prior IPE experience or knowledge; the higher descriptive Cooperation score compared with Partnership; and the proportion of variance not explained by the quantitative model.

The absence of statistical evidence for effect modification by country of work was not treated as a qualitative phenomenon requiring explanation. Instead, the qualitative analysis examined educational, organisational, and relational conditions that could contextualise or extend the pooled quantitative findings.

Initial deductive codes and their theoretical or quantitative sources were documented before systematic coding of the corpus. Text that was not adequately represented by the initial framework was assigned to provisional inductive codes. These codes were compared, refined, and incorporated through iterative revision of the codebook. Each final code was identified as deductive or inductive.

Meaning units could receive up to three codes when multiple assignments were substantively justified. The analysis actively sought dissonant cases that challenged, contradicted, or qualified the dominant interpretation. For the question concerning the importance attributed to IPE, the intensity of the stated position was coded separately from the accompanying explanatory rationale. The complete codebook, including code origins, definitions, inclusion and exclusion criteria, and coding rules, is provided as Supplementary File S1.

#### 2.5.3. Qualitative Rigour and Reflexivity

The initial codebook underwent structured appraisal by two independent experts for relevance, clarity, distinctiveness, and coverage (A.S. and S.C.). Their comments were used to refine code definitions, boundaries between related codes, inclusion and exclusion criteria, and coding instructions before independent coding. The appraisal was used to strengthen the analytical framework and was not treated as psychometric validation of the codebook. The experts’ professional backgrounds, roles, appraisal procedures, and resulting revisions are reported in Supplementary Table S9.

Two coders completed training and pilot coding before independently applying the revised codebook to the complete qualitative corpus. The pilot comprised 20% of the written responses and was selected to include all three open-ended questions and both principal working-country contexts represented in the qualitative corpus. After completing the pilot, the coders reviewed disagreements and clarified code definitions, coding boundaries, and decision rules. They subsequently independently coded the complete corpus using the revised codebook, including recoding the responses included in the pilot.

Agreement on the primary nominal code was evaluated using Cohen’s kappa. Agreement on the ordinal classification of the importance attributed to IPE was evaluated using linear weighted Cohen’s kappa. Agreement for multiple-code assignments was examined separately for each code as binary presence or absence and was supplemented by a set-based Jaccard index comparing the sets of codes assigned by the two coders (R.C. and V.X.). Agreement estimates were calculated using the independent pre-consensus coding. Disagreements were subsequently resolved through discussion, and consensus decisions were documented in the audit trail. Observed agreement estimates are reported in Supplementary Table S9.

Codebook stability and the emergence of additional inductive codes were monitored across sequential blocks of responses. Because the complete available corpus was analysed, this procedure was not interpreted as evidence of sampling saturation and was not used as a sampling-stop rule.

A decision log, version-controlled codebook, analytic memos, and audit trail were maintained throughout the analysis. Reflexive reporting documented the analysts’ professional positions in relation to IPE, their theoretical expectations, their involvement in data collection and analysis, and the potential influence of these positions on directed coding and mixed-methods integration. Independent coding, explicit provision for inductive expansion, dissonant-case analysis, reflexive memoing, and maintenance of an audit trail were used to enhance analytical transparency and reduce confirmation bias.

### 2.6. Mixed-Methods Integration

Integration occurred at the design, analysis, interpretation, and reporting levels [41]. At the design and data-collection level, the optional open-ended questions were embedded within the same survey as the quantitative measures, with priority assigned to the quantitative component. Quantitative measures and written responses were therefore collected concurrently.

At the analytical level, selected quantitative findings informed the qualitative analytical matrix and the explanatory questions applied to the written responses. This procedure represented an analytical linkage between the two components rather than sequential development of qualitative data collection, because the written responses had already been collected before the quantitative findings were available.

At the interpretation and reporting levels, quantitative estimates and qualitative categories were merged using a statistics-by-categories joint display [42]. Qualitative identifiers were not linked to individual quantitative scores; integration was therefore performed at the aggregate-results level rather than through respondent-level or case-based comparison. For each integrated finding, the relationship between the quantitative and qualitative components was classified as convergence, complementarity or expansion, or dissonance. An explicit integrated inference and an interpretive boundary were then formulated.

Qualitative findings were used to interpret, extend, or problematise quantitative patterns. They were not used to validate quantitative estimates, demonstrate mediation, or establish causal mechanisms. Because the qualitative subsample was strongly concentrated among respondents working in Albania, the integrated analysis did not treat the aggregate qualitative categories as geographically representative of the full quantitative sample and did not present qualitative comparisons between Albania and Italy.

### 2.7. Ethical Considerations

The study was conducted in accordance with the Declaration of Helsinki and was approved by the Ethics Committee of the University “Our Lady of Good Counsel”, Tirana, Albania (Protocol No. 110/2026; 6 May 2026). All participants received information concerning the study purpose, procedures, voluntary nature of participation, and data-handling arrangements. Informed consent was obtained electronically before participants accessed the questionnaire. Participants could discontinue questionnaire completion at any time without providing a reason. No directly identifying information was collected. Technical REDCap record identifiers were used exclusively for data-management purposes and could not be used by the analytical team to identify or contact individual participants. Analyses were conducted using a de-identified dataset. Access to the study data was restricted to authorised members of the research team and was governed by the approved study procedures and applicable data-protection requirements.

## 3. Results

### 3.1. Participant Flow and Characteristics

Of the 500 faculty members and educators invited to participate, 445 returned a questionnaire, corresponding to a crude response proportion of 89.0%. The remaining 55 invited individuals did not return a questionnaire; their characteristics and reasons for non-participation were not collected. Country of work was resolved for 442 respondents: 355 worked in Italy and 87 in Albania; three records remained unresolved because the respondents did not indicate their country of work. These records were retained in analyses that did not require country of work and excluded from country-stratified and interaction analyses. The participant and analysis-specific samples are summarised in Supplementary Figure S1.

Participants had a mean age of 54.4 years (SD 12.2) and a mean of 27.1 years of work experience (SD 12.6). The median annual teaching commitment was 100 h (IQR 60–190). Most participants occupied a senior academic role (370/445, 83.1%). Physicians constituted the largest professional group (240/441, 54.4%). Nurses accounted for 28/441 participants (6.3%), other specified health professions for 35/441 (7.9%), and the residual other/unspecified category for 138/441 (31.3%). Because the questionnaire did not include a free-text field for this response option, the underlying professions of these 138 participants could not be recovered or characterised further. Participant characteristics overall and by country of work are presented in Table 1.

### 3.2. Self-Reported Prior IPE Experience, Self-Rated Knowledge, and Perceived Collaboration

Overall, 210 participants (47.2%) reported prior or direct IPE experience. Mean self-rated IPE knowledge was 31.7/100 (SD 25.4) and was similar descriptively in the Albanian and Italian working contexts (31.0 and 31.9, respectively). These context-specific summaries were descriptive and were not interpreted as country effects.

**Figure 1:**
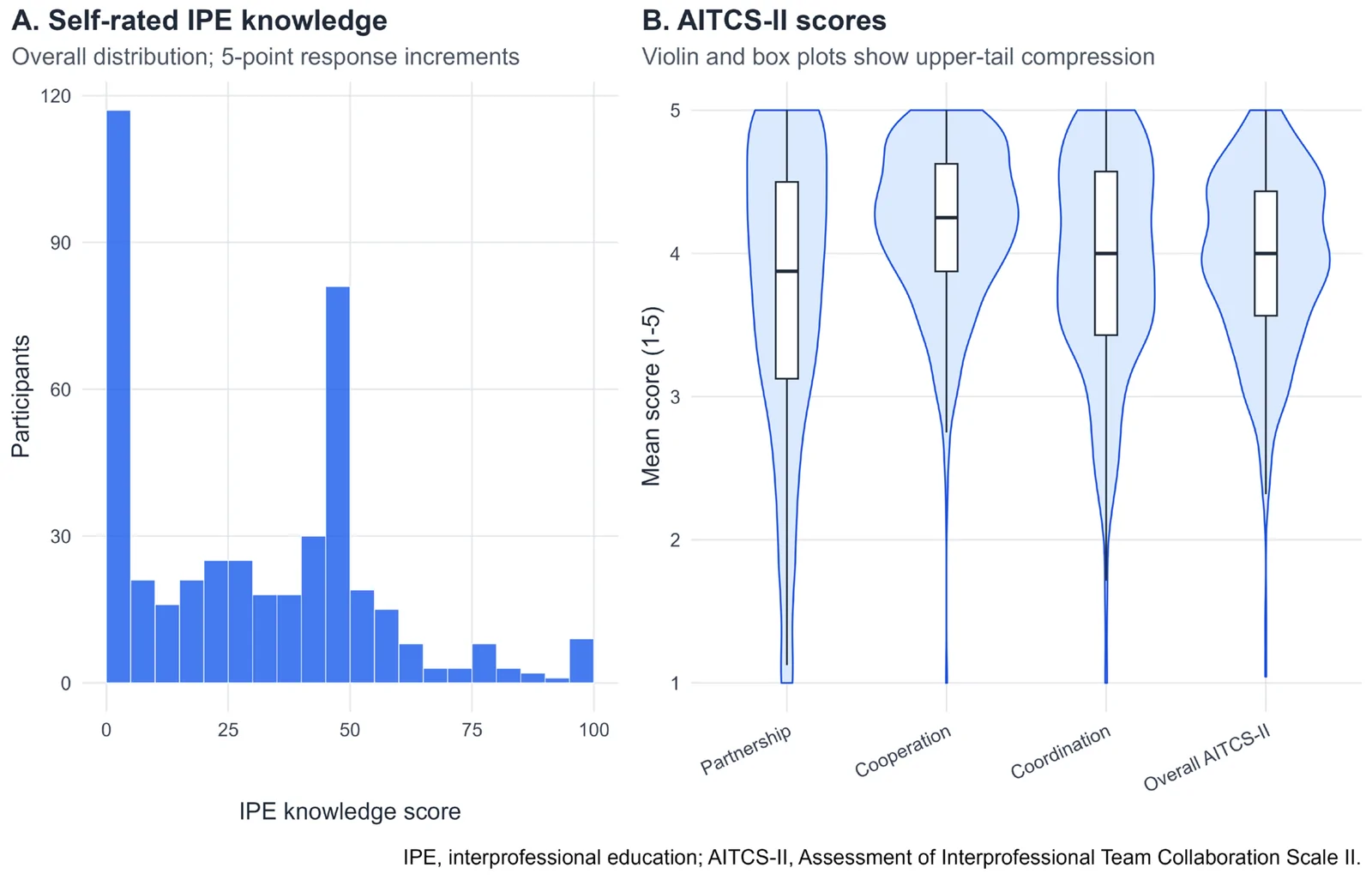
Distributions of self-rated IPE knowledge and perceived interprofessional collaboration. Note: Panel (**A**) shows the distribution of self-rated IPE knowledge scores. Panel (**B**) shows violin and box plots for the three AITCS-II domains and the overall score. AITCS-II, Assessment of Interprofessional Team Collaboration Scale II; IPE, interprofessional education.

The mean AITCS-II overall score was 3.95 (SD 0.61). Cooperation had the highest domain mean (4.20, SD 0.56), followed by Coordination (3.92, SD 0.75) and Partnership (3.74, SD 1.03) (Table 2 and Figure 1). All 23 items showed an upper-end response concentration: the mean proportion selecting category 5 was 35.1%, with item-specific values ranging from 19.1% to 53.9%. Item-level distributions, missingness, floor and ceiling effects, and corrected item-total correlations are reported in Supplementary Table S3.

### 3.3. Preliminary Evidence of Internal Structural Validity and Internal Consistency

Internal consistency was high for the AITCS-II overall score (Cronbach’s alpha = 0.928; ordinal alpha = 0.941) and for each domain. Partnership showed particularly high internal consistency (Cronbach’s alpha = 0.959; ordinal alpha = 0.970), accompanied by a mean inter-item correlation of 0.744, suggesting possible item redundancy rather than an unqualified measurement advantage. Cooperation and Coordination had Cronbach’s alpha values of 0.861 and 0.895, respectively (Table 2; Supplementary Table S4a).

The covariance structure of the item responses was compatible with the hypothesised correlated three-factor model, which showed acceptable fit: chi-square(227) = 306.35, *p* < 0.001, CFI = 0.970, TLI = 0.967, RMSEA = 0.050 (90% CI 0.041–0.060), and SRMR = 0.035. Only one residual correlation exceeded an absolute value of 0.10, and three modification indices were at least 10. These diagnostics were not used to respecify the model. Full reliability estimates, standardised loadings, residual correlations, and modification indices are provided in Supplementary Table S4a–f and Supplementary Figure S3.

Ordinal multigroup measurement invariance could not be estimated because several country-by-item response categories were sparse or empty. In the pragmatic continuous-item MLR sensitivity analysis, absolute changes in CFI and RMSEA were no greater than 0.0023 across the configural, metric, and scalar models. This finding was treated as supportive sensitivity evidence only and not as establishing ordinal measurement invariance.

### 3.4. Ancillary Concordance Analysis

As expected, the two self-reported indicators showed marked concordance. In the common complete-case analytical sample, self-rated knowledge was zero for 86/221 participants without self-reported prior IPE experience (38.9%) and 8/196 participants with prior experience (4.1%). A zero score represented selection of the lowest point on the self-appraisal scale and was not interpreted as evidence that the respondent objectively possessed no knowledge of IPE. The ancillary two-part analysis showed the same general pattern for both the probability of reporting any knowledge and the reported knowledge level among positive responses; complete estimates are provided in Supplementary Table S7e, with the median-regression sensitivity in Supplementary Table S7d. Because absence of prior exposure and absence of perceived familiarity are partly overlapping self-reports, this analysis was not interpreted as evidence of construct validity, educational effectiveness, or causality.

### 3.5. Associations with Perceived Interprofessional Collaboration

Self-rated IPE knowledge had a positive bivariate correlation with the AITCS-II overall score (Spearman’s rho = 0.276); its strongest domain correlation was with Coordination (rho = 0.324). The complete correlation matrix is reported in Supplementary Table S5 and displayed in Supplementary Figure S4.

The primary joint adjusted model included 417 participants. After adjustment for self-rated knowledge and the measured covariates, self-reported prior IPE experience was associated with a 0.32-point higher AITCS-II overall mean score (95% CI 0.18–0.47; *p* < 0.001). Self-rated knowledge also remained associated with the outcome after adjustment for self-reported prior IPE experience and the measured covariates, although the estimate was smaller: each 10-point increase was associated with a 0.03-point higher AITCS-II score (95% CI 0.002–0.058; *p* = 0.035). The joint additive model had an R^2^ of 0.139 and an adjusted R^2^ of 0.120 (Table 3).

Adding the country-by-experience interaction did not increase R-squared (delta R-squared < 0.001; interaction *p* = 0.774). Adding the country-by-knowledge interaction increased R-squared by 0.002; neither this interaction (*p* = 0.428) nor the joint test of both interactions (*p* = 0.677) provided evidence of effect heterogeneity by country of work. Both terms were nevertheless retained in the exploratory final model in accordance with the prespecified hierarchical sequence.

**Figure 2:**
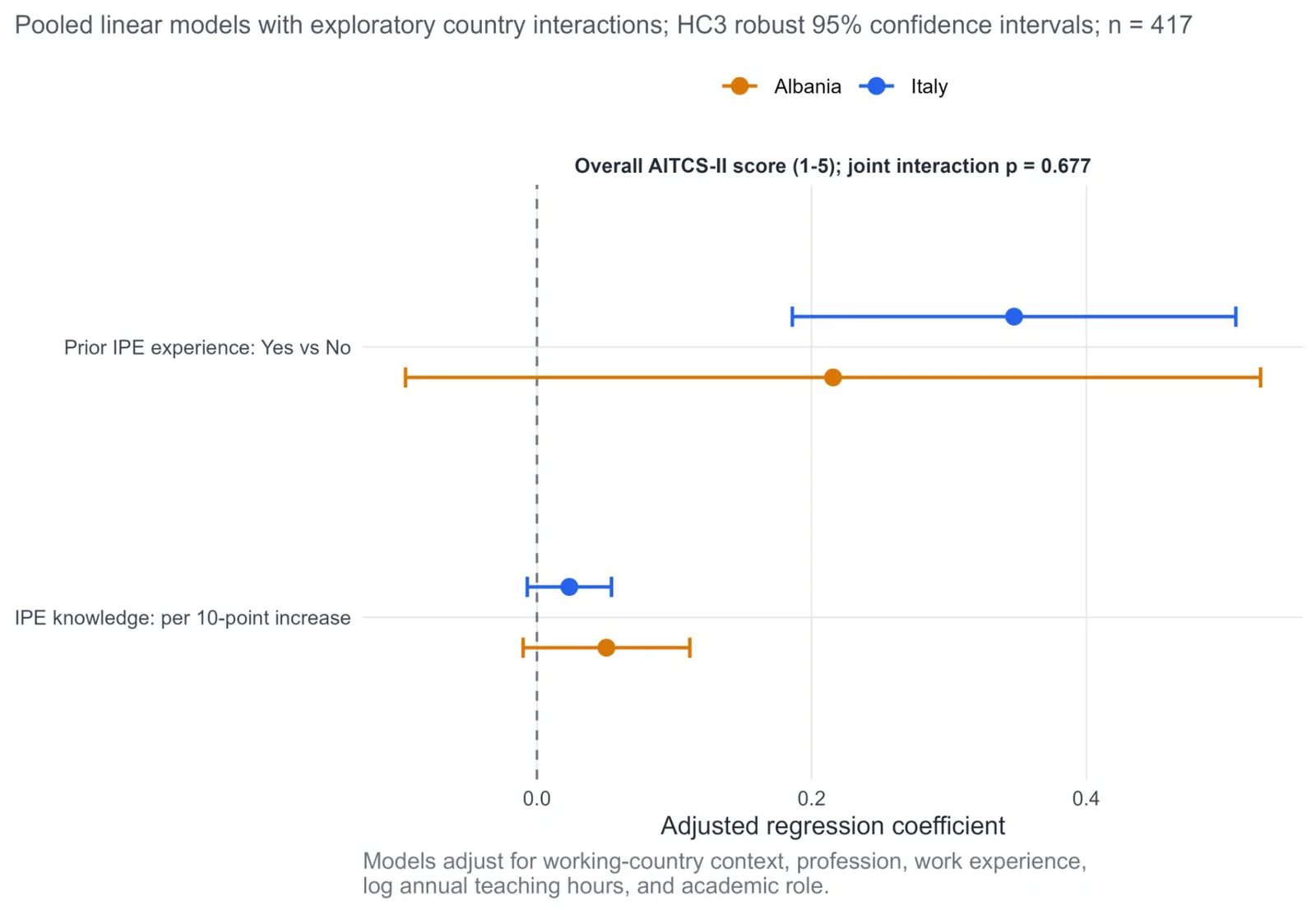
Country-specific adjusted estimates for perceived interprofessional collaboration. Note: Points indicate adjusted regression coefficients and horizontal bars indicate HC3 robust 95% confidence intervals. Models included self-reported prior IPE experience, self-rated IPE knowledge, country of work, profession, work experience, log-transformed annual teaching hours, and academic role (n = 417). The joint interaction test was exploratory (*p* = 0.677). AITCS-II, Assessment of Interprofessional Team Collaboration Scale II; IPE, interprofessional education.

In the final interaction model, the adjusted association for self-reported prior IPE experience was 0.22 points in Albania (95% CI −0.10 to 0.53) and 0.35 points in Italy (95% CI 0.19–0.51). The corresponding estimates per 10-point increase in self-rated knowledge were 0.05 (95% CI −0.01 to 0.11) and 0.02 (95% CI −0.01 to 0.05), respectively (Figure 2). These context-specific estimates were exploratory and were not interpreted as demonstrating different country effects.

### 3.6. Sensitivity Analyses and Model Diagnostics

Self-reported prior IPE experience remained positively associated with Partnership (adjusted difference = 0.39, 95% CI 0.14–0.64; BH-adjusted *p* = 0.004), Cooperation (0.17, 95% CI 0.05–0.30; BH-adjusted *p* = 0.008), and Coordination (0.42, 95% CI 0.25–0.59; BH-adjusted *p* < 0.001). Self-rated knowledge remained associated only with Coordination after false-discovery-rate adjustment (0.05 points per 10-point increase, 95% CI 0.01–0.08; BH-adjusted *p* = 0.029).

Complete-item scoring produced estimates consistent with the primary model. There was no evidence of a quadratic association for self-rated knowledge (*p* = 0.387). Additional adjustment for survey source did not materially change the focal experience or knowledge estimates. However, adding survey source increased the standard error of the country coefficient from 0.079 to 0.146, indicating limited precision in separating the country main effect from the technical recruitment container. Country was therefore retained as a working-context adjustment variable, but its main-effect coefficient was not substantively interpreted.

The final interaction model showed heteroskedasticity (Breusch-Pagan *p* = 0.013), supporting the use of HC3 standard errors; the functional-form test did not indicate misspecification (*p* = 0.765). Collinearity diagnostics were acceptable after accounting for the degrees of freedom of interaction terms, and no observation was excluded solely on the basis of influence statistics. Full coefficients and diagnostics are reported in Supplementary Table S6a–e and Supplementary Figure S5. Sensitivity analyses are reported in Supplementary Table S7a–g.

### 3.7. Qualitative Findings

The embedded qualitative component comprised 341 analysable written responses from 114 participants: 114 responses concerning strategies for strengthening IPE, 113 concerning perceived barriers, and 114 concerning the importance attributed to IPE. The nested qualitative subsample included 86 respondents working in Albania, 25 working in Italy, and three whose country of work remained unresolved. Thus, 86 of the 87 respondents working in Albania contributed analysable qualitative material (98.9%), compared with 25 of the 355 respondents working in Italy (7.0%). Operationally, all analysable Italian qualitative responses came from the “Italia” survey container; no participant recruited through the “Italy2” container provided an analysable response to the optional open-ended section. Reasons for this differential participation were not collected and could therefore not be established. Given this pronounced geographical and survey-source asymmetry, the qualitative findings were analysed for the nested subsample as a whole and not as country comparisons. Because up to three codes could be assigned to each response, category frequencies were not mutually exclusive. Percentages were calculated using the number of valid responses to each open-ended question as the denominator (Supplementary Table S8).

#### 3.7.1. Strategies for Embedding and Strengthening IPE

The proposed strategies primarily concerned curricular and experiential embedding. Revision of curricula and shared teaching across programmes was the most frequent category (38/114, 33.3%). Participants also emphasised structured opportunities for interprofessional encounter (18/114, 15.8%), communication and a shared professional language (18/114, 15.8%), simulation, case-based learning, and other experiential classroom methods (16/114, 14.0%), clinical placements involving real interprofessional teams (13/114, 11.4%), and faculty development and collaboration (13/114, 11.4%). Inductive expansion identified longitudinal and routine integration of IPE, rather than isolated activities, as an additional category (7/114, 6.1%).

One participant described the need to “strengthen interprofessional education during clinical placements, promoting experiences in real teams in which students can participate actively.”

#### 3.7.2. Perceived Barriers to IPE

Barriers spanned cultural, organisational, and resource-related levels. Professional silos and resistance to change were most frequent (21/113, 18.6%), followed by organisational and logistical constraints (18/113, 15.9%), insufficient financial or human resources (17/113, 15.0%), time and workload pressures (16/113, 14.2%), limited motivation or interest (15/113, 13.3%), and inadequate communication or coordination across faculties and departments (13/113, 11.5%).

The interdependence of these barriers was evident in descriptions combining organisational constraints with resource limitations and time pressure. Relational barriers also involved hierarchies, unclear professional boundaries, and resistance to new teaching approaches. As one participant stated, “There are still too many hierarchies in the university setting and difficulties in approaching new teaching methods.”

#### 3.7.3. Importance Attributed to IPE

IPE was described as very important or indispensable in 68/114 responses (59.6%); 15 responses (13.2%) described it as important without an intensifier, five (4.4%) expressed conditional importance, and 26 (22.8%) did not contain an explicit intensity judgement.

The most frequent rationale was preparation for teamwork and professional practice (35/114, 30.7%), followed by patient-centred and comprehensive care (22/114, 19.3%), professional and personal development (18/114, 15.8%), quality and safety of care (17/114, 14.9%), and understanding and respecting professional roles and competencies (13/114, 11.4%). Inductive analysis additionally identified management of complexity and integration of multiple perspectives (11/114, 9.6%). This rationale was illustrated by the observation that “medicine has become too specialised and often requires a broader perspective.” Complete category frequencies, including the separate intensity and rationale axes for open answers to question 3, are reported in Supplementary Table S10.

#### 3.7.4. Qualitative Rigour

Two experts independently appraised the initial codebook for relevance, clarity, distinctiveness, and coverage. Both experts judged all 49 a priori categories to be relevant and sufficiently clear; their comments were used to refine category boundaries and coding instructions before independent coding.

Primary-code agreement between the two independent coders was 93.0% for strategies (kappa = 0.920), 93.8% for barriers (kappa = 0.933), and 93.9% for importance rationales (kappa = 0.929). Mean set-based Jaccard indices for multiple-code assignment ranged from 0.978 to 0.982. For the ordinal importance classification, exact agreement was 97.4% and linear weighted kappa was 0.983. Full expert-appraisal, agreement, codebook-stability, and audit information is reported in Supplementary Table S9; the complete codebook is provided as Supplementary File S1.

### 3.8. Mixed-Methods Integration

The joint display integrates pooled quantitative estimates with aggregate qualitative categories from the nested voluntary-response subsample. Because respondents working in Albania constituted 75.4% of the qualitative subsample and had a substantially higher qualitative participation proportion than respondents working in Italy, the qualitative component may disproportionately reflect the Albanian institutional and cultural context. The integrated findings should therefore not be interpreted as geographically representative of the overall quantitative sample. Integration showed convergence, expansion, and limited productive dissonance across the two components (Table 4). First, the robust association observed for self-reported prior IPE experience, alongside the smaller and less precisely estimated association for self-rated knowledge, was expanded by participants’ emphasis on simulation, case-based learning, clinical placement, repeated interaction, and longitudinal curricular integration. This pattern suggests that translation into collaborative practice may depend on applied, repeated, and contextually embedded learning, without demonstrating mediation or causality.

Second, at the aggregate-results level, accounts of professional silos, status hierarchies, unclear role boundaries, and weak coordination across organisational units provided complementary context for interpreting the numerically higher Cooperation score and lower Partnership score. These accounts are compatible with the possibility that operational cooperation may occur without fully symmetrical decision-making, reciprocal recognition, or shared responsibility. However, qualitative responses could not be linked to individual AITCS-II scores; the analysis therefore does not show that participants reporting these barriers were those with lower Partnership scores or directly explain the observed domain pattern.

Third, leadership, curricular rigidity, scheduling, resources, faculty development, communication across organisational units, and professional culture emerged as plausible determinants not represented in the quantitative model. These categories expand the explanatory field around the model’s adjusted R^2^ of 0.120 and identify candidate contextual variables for future research.

Finally, at the sample level, respondents strongly valued IPE for teamwork, comprehensive care, quality and safety, and the management of complexity, despite the overall prevalence of formal IPE exposure being below 50% and mean self-rated knowledge being modest. This productive dissonance is consistent with the possibility that collaborative orientations may also develop through professional practice or informal learning not identified by respondents as formal IPE. The available data did not establish this pathway at the individual level. No qualitative country comparison was used to explain the null interaction tests.

## 4. Discussion

The main finding of this study is that self-reported prior IPE experience and self-rated IPE knowledge were related but non-interchangeable in their associations with perceived interprofessional collaboration. In the joint adjusted model, self-reported prior IPE experience showed a clearer and more consistent adjusted association with the overall AITCS-II score, whereas self-rated knowledge showed a smaller adjusted association. Experience was also associated with all three AITCS-II domains, while the adjusted association of knowledge remained statistically supported only for Coordination after adjustment for multiple testing. The exploratory interaction analyses did not provide evidence that these associations differed between the Albanian and Italian working contexts. However, the null interaction tests should not be interpreted as demonstrating contextual equivalence.

The specific contribution of this study does not lie in establishing the general proposition that educational exposure and collaboration are related. Rather, it refines how this relationship should be interpreted among faculty members and educators. First, by examining self-reported prior IPE experience and single-item self-rated IPE knowledge simultaneously, the study indicates that, as operationalised here, these commonly conflated indicators provide non-interchangeable information; importantly, this does not demonstrate that experience is inherently more important than knowledge. Second, the domain-level findings show that a favourable overall collaboration score may conceal an uneven profile in which operational Cooperation is perceived more strongly than the reciprocal participation and shared responsibility represented by Partnership. Third, aggregate mixed-methods integration extends the quantitative associations by identifying educational and organisational conditions that may facilitate or constrain the translation of IPE into collaborative practice, including opportunities for repeated application, faculty preparation, professional hierarchies, curricular organisation, resources, and cross-unit governance. These findings generate more specific hypotheses about educational implementation and transfer, rather than establishing a causal mechanism.

### 4.1. Experience, Knowledge, and Translation into Collaborative Practice

The clearer and more consistent association observed for self-reported prior IPE experience is consistent with evidence that IPE can improve teamwork-related competencies and collaborative practice, particularly when learning is experiential and connected to authentic professional situations [7,9,11]. The present findings extend this literature by showing that reported experience remained associated with perceived collaboration after self-rated knowledge and relevant professional and academic characteristics were considered simultaneously. On the 1–5 AITCS-II scale, the adjusted 0.32-point difference associated with self-reported prior IPE experience corresponded to approximately half of the observed standard deviation of the overall score (SD = 0.61), with a 95% CI ranging from 0.18 to 0.47 points. By comparison, a 10-point increase in the single-item knowledge rating corresponded to 0.03 points; an increase equal to one observed standard deviation of knowledge (25.4 points) would correspond to approximately 0.08 points on the outcome scale. These comparisons describe the magnitude of the associations but do not establish their practical or educational importance. However, this adjusted association should not be interpreted as an independent educational effect. Institutional characteristics, previous formal teamwork training, clinical versus predominantly academic employment, leadership responsibilities, programme type, and current participation in interprofessional teams may have been related to both prior IPE exposure and perceived collaboration but were not available in sufficient detail for adjustment. Residual confounding therefore remains plausible. In addition, prior experience was elicited through a broad binary question without an operational definition of qualifying IPE activities. It may have encompassed formal or informal experiences encountered as a student, faculty member, facilitator, or healthcare professional and did not capture timing, dose, duration, format, pedagogical quality, professional mix, facilitation, or degree of workplace integration. It may therefore also reflect access to better-developed interprofessional environments or a pre-existing orientation towards collaborative practice.

The smaller association observed for self-rated IPE knowledge should be interpreted in light of how this construct was measured. The single author-developed item captured respondents’ subjective appraisal of their familiarity with and understanding of IPE rather than objectively assessed knowledge. Its marked concentration at zero and heaping at multiples of 5 or 10 further suggest that respondents may have used the 0–100 scale as a broad confidence or familiarity rating rather than as a finely graded estimate of knowledge. As with self-assessment more broadly, ratings may also have been influenced by differences in metacognitive calibration: respondents with limited familiarity may have been less able to recognise gaps in their understanding, whereas more knowledgeable respondents may have applied more demanding internal standards. Consequently, the comparison between the two focal indicators does not demonstrate that experience is inherently more important than knowledge. It shows more narrowly that self-reported prior IPE experience had a clearer and more consistent association with perceived collaboration than did this single-item measure of self-perceived knowledge in the present sample. Differences in measurement scale, precision, and construct coverage may also have contributed to the contrast between their associations [43]. The association of the knowledge rating was most evident for Coordination, although only this domain remained statistically supported after multiplicity adjustment. This pattern may indicate that perceived familiarity with IPE is related to how respondents recognise the organisation of roles, information, and activities; however, it should not be interpreted as establishing a specific effect of knowledge on coordination. Partnership, however, also requires reciprocity, shared responsibility, and participation in decision-making, i.e., processes that may be less readily acquired through declarative knowledge alone.

The ancillary experience–knowledge analysis showed expected concordance between two related self-reported indicators but was not a validation analysis [33]. The relationship was partly definitional because respondents who reported no prior encounter with IPE were also more likely to report no familiarity with it. Accordingly, neither the zero-score comparison nor the two-part model should be interpreted as evidence of construct validity, an educational gain, or programme effectiveness.

### 4.2. Cooperation, Coordination, and Partnership

The domain pattern provides a second substantive contribution. Cooperation was rated highest, whereas Partnership was rated lowest and was substantially more variable. This distinction accords with critiques that generic descriptions of teamwork can obscure differences between operational cooperation and more demanding forms of shared power, reciprocal recognition, and collective decision-making [12]. Professionals may work together effectively at the task level while continuing to experience status asymmetries, uncertain professional boundaries, or limited participation in decisions [44].

The overall collaboration score was closely similar to that reported by Pun et al. [24] among 3586 professionals from 68 intensive care units in the United States: 3.95 (SD 0.61) in the present study compared with 3.92 (SD 0.64). The domain profiles, however, differed. Pun et al. [24] reported Partnership/shared decision-making as the highest-rated domain and Coordination as the lowest, whereas Cooperation was highest and Partnership lowest in the present sample. This comparison is descriptive rather than direct because Pun et al. [24] studied practising intensive care professionals and used the original 37-item AITCS, in which Partnership and shared decision-making formed a combined domain, whereas the present study used the revised 23-item AITCS-II. Nevertheless, the similarity of the overall scores alongside the contrasting domain profiles reinforces the value of examining the composition of collaboration rather than relying on the total score alone.

At the aggregate-results level, the qualitative accounts provide complementary context for this interpretation but do not directly test it. Participants described professional silos, hierarchies, unclear roles, and weak coordination across faculties and departments, while also calling for a shared professional language and structured opportunities for interaction. Because written responses were not linked to individual AITCS-II scores, these accounts cannot be attributed specifically to participants with lower Partnership scores. These accounts are consistent with evidence that role clarity, communication, familiarity, leadership, and organisational support shape interprofessional teamwork [45,46,47]. Accordingly, programmes should not assume that bringing professions together or coordinating activities is sufficient to develop partnership. Educational designs may need to address decision-making, professional power, reciprocal accountability, and negotiation of roles explicitly [46].

The domain pattern must also be considered alongside the marked ceiling effect. Every AITCS-II item had more than 15% of responses in the highest category, and Cooperation showed the greatest upper-end concentration. This restricted outcome variability may have reduced the instrument’s ability to discriminate among respondents with relatively favourable perceptions and may have contributed to underestimation of the observed associations with self-reported prior IPE experience and the single-item knowledge rating, particularly for Cooperation. It also makes an unvalidated threshold such as a mean score of 4 or higher poorly suited to classifying collaboration as adequate, because that threshold lies within the compressed portion of the distribution [6,22]. Continuous scores and domain-specific profiles are therefore more informative in this sample.

### 4.3. Organisational Conditions and the Unexplained Variance

The adjusted quantitative model explained approximately 12% of the variance in the overall AITCS-II score, meaning that most between-participant variation remained unexplained. This does not itself establish residual confounding, but it is consistent with collaboration being shaped by determinants not fully represented by the measured individual, professional, and educational variables. The qualitative component identified leadership, curricular organisation, scheduling, workload, human and financial resources, faculty development, cross-unit communication, and professional culture as plausible additional conditions. Similar multilevel determinants recur in implementation research on IPE, which emphasises the interaction of teaching practices, institutional structures, faculty readiness, and broader professional systems [48,49].

The value of the qualitative findings lies in expanding the explanatory field around the regression model, not in attributing portions of residual variance to particular categories [50]. Category frequencies indicate salience within the voluntary-response subsample; they do not estimate effect sizes, population prevalence, or causal importance. Future quantitative studies could measure these contextual constructs explicitly and examine whether they confound, mediate, or modify the relationship between IPE exposure and collaborative practice. These qualitative categories should not, however, be interpreted as context-neutral explanations of the pooled quantitative findings. Because nearly all respondents working in Albania but only a small minority of those working in Italy contributed written responses, the identified organisational and cultural conditions may disproportionately reflect the Albanian context, while Italian experiences were underrepresented. Their contribution is therefore to identify plausible contextual conditions and generate hypotheses rather than to characterise their distribution across the full sample or across working-country contexts.

At the sample level, respondents strongly endorsed IPE despite the prevalence of formal IPE exposure being below 50% and mean self-rated knowledge being modest. This productive dissonance is consistent with the possibility that collaborative values may also develop through professional experience, informal learning, or participation in multidisciplinary work that respondents do not identify as formal IPE [51]. Conversely, valuing IPE does not ensure that institutions provide the time, resources, governance, or psychological safety required to enact it [52]. The available data did not establish these pathways at the individual level, but the distinction may help explain why positive attitudes towards IPE can coexist with persistent implementation barriers.

### 4.4. Working-Context Comparisons

The exploratory interaction tests did not show statistical evidence that the associations of experience or knowledge with perceived collaboration differed by country of work. This finding offers no basis for claiming that the two contexts are equivalent. The Albanian group was smaller, the interaction analyses were not separately powered, and the context-specific confidence intervals were correspondingly imprecise. In addition, definitive ordinal measurement invariance could not be established. The continuous-item robust maximum-likelihood sensitivity analysis showed only small changes in fit across configural, metric, and scalar models, but this pragmatic result cannot replace an ordinal invariance analysis using the original five response categories.

Country of work should also be distinguished from nationality, language, and survey source. It represented the respondent’s current working context, including participants who may work outside their country of origin. Respondents could select the survey language, and the Albanian component included one Italian-speaking university and two institutions delivering programmes in English. The Albanian working context was therefore linguistically and institutionally transnational rather than a homogeneous proxy for nationality or questionnaire language. Larger, more balanced samples, common recruitment procedures, and adequate representation of every response category would be required for confirmatory contextual comparisons.

### 4.5. Measurement Considerations

The CFA indicated that the covariance structure of the responses was compatible with the hypothesised correlated three-domain structure of the AITCS-II [21,22]. Internal consistency estimates were high for the overall and domain scores, although the very high alpha and mean inter-item correlation for Partnership suggested possible item redundancy rather than an unequivocal measurement advantage. Together with the ceiling effect, this finding indicates that some items may provide limited additional discrimination in a sample of experienced faculty and educators.

Taken together, these findings provide preliminary, sample-specific evidence of internal structural validity and internal consistency; they do not constitute validation of the AITCS-II for faculty or educator populations or establish content validity for this context of use. Confirmatory factor analysis evaluates whether the observed relationships among items are compatible with a specified measurement structure, but it cannot determine whether the items adequately represent collaboration across educational, organisational, and clinical settings. Nor can it establish that the construct has the same meaning as it does among practitioners responding with reference to a clearly bounded healthcare team [53].

The administered AITCS-II instructions framed teamwork as regular interaction among health professionals for the purpose of providing patient care. However, participants were not asked to identify a specific healthcare team or to report whether their answers were based on clinical practice, working relationships as educators, or overlapping professional roles. The frame of reference actually used by each respondent therefore could not be verified, and some between-participant variation may reflect differences in the professional relationships considered when answering the items. Further research should therefore examine content relevance and interpretation among faculty and educators, for example through cognitive interviewing and target-population appraisal, and should evaluate the instrument separately across clearly specified educational, organisational, and clinical frames of reference.

### 4.6. Implications for Education, Organisations, and Research

These findings support further consideration of longitudinal curricular embedding and repeated, facilitated opportunities for learners to work through authentic cases, simulations, and clinical or community-based problems with other professions [54,55]. Educational strategies may also benefit from learning objectives that extend beyond generic teamwork to include role negotiation, shared decision-making, management of professional disagreement, and equitable participation. Faculty development may be particularly relevant because educators both design IPE and model the professional relationships that learners are expected to adopt [56].

At the organisational level, protected time, aligned schedules, cross-programme governance, adequate staffing and resources, and institutional recognition of interprofessional teaching may help support sustainable implementation. These considerations are consistent with the implementation determinants identified in previous reviews and with the barriers described by respondents in the present study [13]. Their effectiveness was not evaluated in this study and should be examined prospectively.

Future evaluations should document the dose, duration, professional composition, facilitation, authenticity, and continuity of IPE rather than recording exposure only as present or absent. Prospective and longitudinal designs are needed to examine change over time and distinguish educational selection from transfer into practice. Outcomes should combine self-report with behavioural observation, peer or learner assessment, organisational indicators, and, where appropriate, patient- or system-level measures. Claims about patient benefit should remain cautious because direct evidence linking interprofessional collaboration to patient-reported outcomes remains heterogeneous [57].

### 4.7. Strengths and Limitations

This study has several strengths. It included a relatively large, multicentre sample of health professions faculty and educators from two working-country contexts and distinguished self-reported prior IPE experience from self-rated knowledge. The analytical strategy was theory-informed and specified before the final modelling stage, used robust standard errors, retained exploratory interactions without automated *p*-value-driven selection, and incorporated domain-specific, non-linearity, scoring, and survey-source sensitivity analyses. Preliminary examination of internal structural validity and internal consistency preceded substantive modelling. Finally, the quantitative-dominant mixed-methods design supported integration at the aggregate-results level through directed qualitative content analysis with inductive expansion and a statistics-by-categories joint display.

Several limitations constrain interpretation. First, the cross-sectional design cannot establish temporality or causality. Self-reported prior IPE experience, self-rated IPE knowledge, and perceived collaboration were measured within the same survey and may have been affected by recall bias, social-desirability bias, and common-source or common-method bias. Moreover, the AITCS-II captured perceived collaboration rather than observed collaborative behaviour. Although the administered AITCS-II instructions framed teamwork in relation to interactions among health professionals providing patient care, the survey did not record the specific team or setting used as the referent. Nor did it establish whether respondents drew primarily on clinical practice, their working relationships as educators, or overlapping academic, organisational, and clinical roles. This uncertainty may have introduced heterogeneity in the meaning of the scores and limits their interpretation as assessments of collaboration within a uniform type of healthcare team. The CFA and reliability analyses provided only preliminary, sample-specific evidence of internal structural validity and internal consistency; they cannot determine whether the items had equivalent content relevance or meaning across these different contexts. The knowledge indicator was a single author-developed self-rating rather than a validated multidimensional instrument or objective knowledge assessment. It may also have been influenced by differences in respondents’ ability to recognise gaps in their own understanding. Its concentration at the lower bound of zero and heaping at multiples of 5 or 10 may indicate heterogeneous use of the response scale, limiting the interpretability of its coefficient and direct comparison with the binary experience indicator. The observed association therefore pertains specifically to self-perceived IPE knowledge as measured in this study and should not be generalised to IPE knowledge as a broader construct. Likewise, self-reported prior IPE experience was measured using a broad binary item without an operational definition of qualifying activities. It did not distinguish whether exposure occurred as a student, faculty member, facilitator, or healthcare professional, whether it was formal or informal, or its timing, dose, duration, format, quality, or fidelity. Heterogeneous interpretation of the item may therefore have introduced exposure misclassification. Differences in measurement scale, precision, and construct coverage may therefore have contributed to the contrast between the two associations. Residual confounding also remains plausible because adjustment was limited to the covariates measured in the survey. Unmeasured or insufficiently characterised institutional conditions, previous formal teamwork training, clinical versus predominantly academic employment, leadership responsibilities, programme type, and current participation in interprofessional teams may have been related to both prior IPE exposure and perceived collaboration. The adjusted coefficient for self-reported prior IPE experience should therefore be interpreted as an association conditional on the measured covariates rather than as an independent educational effect. Finally, the high AITCS-II scores and pervasive ceiling effect may have restricted outcome variability and attenuated the observed associations.

Second, the convenience sample was dominated by senior academics and physicians, while almost one third of participants reported professions classified as “other” or unspecified and could not be categorised more precisely. Consequently, adjustment for professional background was necessarily coarse and may not have captured differences among the professions grouped within this residual category. This composition limits generalisability to the broader population of health professions faculty and educators, particularly junior faculty, clinical educators, nurses, allied health professionals, tutors, and other less represented educational roles. Because senior academics and physicians may occupy positions of greater formal or informal authority, their overrepresentation may have introduced a power-related compositional bias. The aggregate scores and qualitative accounts may therefore disproportionately reflect the perspectives of respondents with greater influence over curricula, organisational decisions, and interprofessional working relationships, while underrepresenting experiences of limited voice, exclusion, or hierarchy among junior faculty and less represented professional groups. The direction and magnitude of this potential bias cannot be determined from the available data. Although all individuals identified as eligible on the accessible institutional lists were invited, these lists constituted a convenience-based institutional sampling frame rather than a population register and may not have covered the broader population of health professions faculty and educators. The crude questionnaire-return proportion of 89.0% applies only to the 500 individuals invited from these lists. It may have been facilitated by repeated reminders and personal encouragement from local investigators and academic or programme coordinators, but it does not establish representativeness or eliminate the possibility of institutional and self-selection bias. No information was collected on the characteristics, confirmed eligibility, or reasons for non-participation of the 55 individuals who did not return a questionnaire, precluding direct assessment of non-response bias. Complete-case modelling may also have introduced selection bias if missingness was related to unobserved participant characteristics.

Third, contextual inference was limited by the smaller Albanian sample, three unresolved working-context records, the structural overlap between the Italian context and the survey source, and the inability to estimate definitive ordinal measurement invariance. Neither the null interactions nor the descriptive country-specific estimates establish equivalence or difference between the two working-country contexts.

Finally, the qualitative component consisted of brief optional written responses from 114 participants rather than interviews or focus groups. Qualitative participation differed markedly by working context: 86 of 87 respondents working in Albania contributed analysable text (98.9%), compared with 25 of 355 respondents working in Italy (7.0%). Although the open-ended questions were available in all questionnaire containers and no source was excluded a priori, no participant recruited through the “Italy2” container provided analysable optional text. Reasons for participation or non-participation in the open-ended section were not collected. The aggregate qualitative categories may therefore disproportionately reflect the Albanian institutional and cultural context and may also overrepresent participants who were especially engaged with IPE. Analysis at the manifest level supported systematic categorisation but limited exploration of implicit meanings and institutional processes. The directed approach carried a risk of confirming the quantitative framework, although inductive expansion, independent coding, consensus procedures, reflexive practices, and an audit trail were used to mitigate this risk. Because the complete available corpus was analysed, saturation was neither used as a sampling-stop criterion nor claimed as an established property of the analysis. Integration was performed at the level of aggregate statistics and qualitative categories; qualitative identifiers were not linked to individual quantitative scores, precluding respondent-level comparisons of extreme or discordant cases. The qualitative findings should therefore be interpreted as contextualising, complementary, and hypothesis-generating rather than as explanations of individual quantitative scores, representative cross-context comparisons, or confirmation of the quantitative associations.

## 5. Conclusions

Among health professions faculty and educators, self-reported prior IPE experience showed a clearer and more consistent association with perceived interprofessional collaboration than did the single-item measure of self-rated IPE knowledge. This comparison should not be interpreted as showing that experience is inherently more important than knowledge, because the two constructs were measured differently and knowledge was not assessed objectively. Cooperation was perceived more favourably than Partnership, while qualitative accounts highlighted professional hierarchies, role boundaries, and organisational constraints that may limit shared decision-making and reciprocal responsibility. Together with the broader literature, participants’ accounts support further consideration of applied and contextually embedded IPE, accompanied by faculty development, cross-programme governance, and organisational support; however, these strategies were not evaluated as interventions in this cross-sectional study. The findings do not establish causality, actual collaborative behaviour, or equivalence between working-country contexts. Prospective studies using more balanced samples, detailed measures of IPE exposure, validated or objective knowledge assessments, behavioural outcomes, and definitive ordinal measurement-invariance testing are needed to examine whether and how IPE relates to collaborative practice across contexts.

## Figures and Tables

**Table 1:** Participant characteristics overall and by country of work.

Characteristic	Overall	Albania	Italy
Participants, n	445	87	355
Age, years	54.4 (12.2)	52.7 (13.6)	55.0 (11.8)
Work experience, years	27.1 (12.6)	25.3 (13.8)	27.6 (12.3)
Annual teaching hours	100.0 [60.0–190.0]	100.0 [50.0–150.0]	100.0 [60.0–190.0]
Prior/direct IPE experience, yes	210 (47.2%)	36 (41.4%)	173 (48.7%)
Academic role, senior	370 (83.1%)	69 (79.3%)	300 (84.5%)
Profession: physician	240 (54.4%)	50 (57.5%)	188 (53.6%)
Profession: nurse	28 (6.3%)	5 (5.7%)	23 (6.6%)
Profession: other specified health profession	35 (7.9%)	7 (8.0%)	28 (8.0%)
Profession: other/unspecified	138 (31.3%)	25 (28.7%)	112 (31.9%)
Education: PhD	311 (70.7%)	61 (71.8%)	247 (70.2%)
Survey source 2 (Central and Southern Italy)	330 (74.2%)	0 (0.0%)	330 (93.0%)

Note: Values are mean (SD), median [IQR], or n (%). Percentages use non-missing denominators: age, work experience and profession, n = 441; education, n = 440; teaching hours, n = 431; academic role and self-reported prior IPE experience, n = 445. Country columns exclude three respondents whose country of work was not specified. Other specified health profession comprises physiotherapists (n = 25), psychologists (n = 7) and midwives (n = 3), grouped because each contained fewer than 10 respondents. Other/unspecified corresponds to the residual “other” response option; no free-text field was provided, so the underlying professions are not recoverable. Survey source 2 (Central and Southern Italy) is the second Italian data-collection container (93.0% of Italian respondents). Age and work experience were collinear (r = 0.94), so only work experience was retained in the models; teaching hours were right-skewed and log-transformed for modelling. No statistical tests were performed between country columns.

**Table 2:** Self-reported prior IPE experience, self-rated knowledge, and AITCS-II summary.

Measure	Overall	Albania	Italy	Cronbach Alpha	Ordinal Alpha	Composite Reliability	Item Category 5, Mean (Range)
Prior/direct IPE experience, yes	210 (47.2%)	36 (41.4%)	173 (48.7%)	–	–	–	–
Self-rated IPE knowledge (0–100)	31.7 (25.4)	31.0 (28.8)	31.9 (24.3)	–	–	–	–
Partnership	3.74 (1.03)	3.85 (1.12)	3.71 (1.00)	0.959	0.970	0.970	32.4% (23.6–36.1)
Cooperation	4.20 (0.56)	4.26 (0.64)	4.18 (0.53)	0.861	0.897	0.898	40.1% (27.2–53.9)
Coordination	3.92 (0.75)	3.98 (0.82)	3.91 (0.73)	0.895	0.921	0.922	32.6% (19.1–38.2)
Overall AITCS-II	3.95 (0.61)	4.03 (0.71)	3.94 (0.58)	0.928	0.941		35.1% (19.1–53.9)

Note: Values are n (%) or mean (SD), as appropriate. Item category 5 is the mean proportion (range) selecting the highest response category across items. AITCS-II, Assessment of Interprofessional Team Collaboration Scale II; IPE, interprofessional education.

**Table 3:** Primary associations and exploratory country interactions for the overall AITCS-II score.

Model	Term	Estimate (95% CI)	*p*-Value	∆R-Squared	R-Squared	Adjusted R-Squared
Model 1. Joint additive model	Self-reported prior IPE experience: yes vs. no	0.322 (0.177 to 0.466)	<0.001	–	0.139	0.120
Model 1. Joint additive model	Self-rated IPE knowledge: per 10-point increase	0.030 (0.002 to 0.058)	0.035	–	0.139	0.120
Model 2. Model 1 + country × experience	Country of work x self-reported prior IPE experience	0.043 (−0.250 to 0.336)	0.774	<0.001	0.139	0.118
Model 3. Model 2 + country × knowledge (final)	Country of work × self-reported prior IPE experience	0.132 (−0.213 to 0.477)	0.453	0.002	0.141	0.117
Model 3. Model 2 + country × knowledge (final)	Country of work × self-rated IPE knowledge	−0.027 (−0.094 to 0.040)	0.428	0.002	0.141	0.117
Model 3. Model 2 + country × knowledge (final)	Joint 2-degree-of-freedom test of both country interactions	Not applicable	0.677	0.002	0.141	0.117

Note: Estimates are adjusted linear-regression coefficients on the AITCS-II mean-score scale (1–5) with HC3 robust 95% confidence intervals (n = 417). All models included self-reported prior IPE experience, self-rated IPE knowledge, country of work, profession, work experience, log-transformed annual teaching hours, and academic role. Interaction coefficients express the difference in the corresponding association for the Italian relative to the Albanian working context; ΔR^2^ refers to the change from the preceding model in the sequence. The interaction sequence was prespecified and exploratory, was not separately powered for cross-context comparison, and both interaction terms were retained in the final model irrespective of statistical significance. Complete coefficients are reported in Supplementary Table S6a and the interaction tests in Supplementary Table S6d. AITCS-II, Assessment of Interprofessional Team Collaboration Scale II; CI, confidence interval; IPE, interprofessional education.

**Table 4:** Mixed-methods joint display.

Quantitative Results	Qualitative Finding	Relationship	Integrated Inference	Interpretive Boundary
Self-reported prior IPE experience showed a clearer association with the AITCS-II overall score than self-rated knowledge.	Respondents prioritised simulation, case-based learning, clinical placements, repeated encounters, and longitudinal curricular integration.	Convergence and expansion	Participants’ accounts identified applied, repeated, and contextually embedded learning as potentially relevant to connecting IPE exposure with collaborative working relationships.	Aggregate complementarity only; individual-level linkage, mediation, comparative educational effects, and causality were not tested.
Cooperation had the highest domain mean, whereas Partnership had the lowest.	Professional silos, status hierarchies, unclear role boundaries, and weak cross-unit coordination were described.	Complementarity and expansion	The qualitative accounts provide contextual support for the possibility that operational cooperation may coexist with barriers to symmetrical decision-making, reciprocal recognition, and fully shared responsibility.	Qualitative responses were not linked to individual AITCS-II scores; respondents reporting these barriers cannot be identified as those with lower Partnership scores, and the accounts do not directly explain the domain-score pattern.
The primary additive model had an adjusted R-squared of 0.120.	Leadership, curricular rigidity, scheduling, resources, faculty development, and professional culture emerged as salient conditions.	Expansion	Important contextual determinants of perceived collaboration were not represented in the quantitative model.	Qualitative frequency indicates salience, not effect size or causal importance.
Country interaction tests did not support heterogeneous associations.	Shared barriers and contextually salient conditions were described, but the qualitative subsample was geographically unbalanced.	Complementarity without confirmation of heterogeneity	Context may shape opportunities and meanings, but the data do not demonstrate different country-specific association coefficients.	No representative qualitative country comparison was conducted.

Note: Integrated inferences combine pooled quantitative estimates with aggregate qualitative categories from the nested voluntary-response subsample. Qualitative identifiers were not linked to individual quantitative scores; the joint display therefore represents aggregate statistics-by-categories integration rather than respondent-level or case-based comparison. Respondents working in Albania constituted 75.4% of the qualitative subsample, and qualitative participation differed markedly between the Albanian and Italian working contexts. The qualitative categories may therefore disproportionately reflect the Albanian institutional and cultural context and should not be interpreted as geographically representative of the overall quantitative sample. Qualitative frequencies indicate salience within the voluntary-response subsample and do not represent population prevalence, effect sizes, mediation, or causal mechanisms. AITCS-II, Assessment of Interprofessional Team Collaboration Scale II; IPE, interprofessional education.

## Data Availability

The deidentified quantitative dataset supporting the findings of this study is openly available in Figshare: Caruso, R. Deidentified Quantitative Dataset on Interprofessional Education and Perceived Interprofessional Collaboration among Health Professions Faculty and Educators; 2026; 85,231 bytes. Available at: https://figshare.com/articles/dataset/Deidentified_Quantitative_Dataset_on_Interprofessional_Education_and_Perceived_Interprofessional_Collaboration_among_Health_Professions_Faculty_and_Educators/33106577?file=67069610, accessed on 30 July 2026. The qualitative free-text responses are not publicly available because they may contain contextual information that could increase the risk of participant identification. Access to these data may be considered by the corresponding author upon reasonable request, subject to ethical approval and applicable data-protection requirements.

## Supplementary Materials

The following supporting information can be downloaded at: https://www.mdpi.com/article/10.3390/healthcare14162649/s1, Table S1: Variable definitions, response formats, scoring rules, and analytical roles; Table S2: Data-quality and missingness summary; Table S3: AITCS-II item-level statistics; Table S4a–f: Preliminary psychometric analyses; Table S5: Spearman correlation matrix; Table S6a–e: Primary regression models and diagnostics; Table S7a–g: Sensitivity analyses; Tables S8–S10: Qualitative subsample characteristics, analytical rigour, and complete category frequencies; Figures S1–S5: Participant flow, missingness, psychometric findings, correlation matrix, and regression diagnostics; Supplementary File S1: Qualitative codebook; Supplementary File S2: Completed STROBE reporting checklist; Supplementary File S3: Completed SRQR reporting checklist; Supplementary File S4: Completed GRAMMS reporting checklist.

## Abbreviations

The following abbreviations are used in this manuscript:

IPE	Interprofessional education
AITCS-II	Assessment of Interprofessional Team Collaboration Scale II
I-AITCS II	Italian Assessment of Interprofessional Team Collaboration Scale II
REDCap	Research Electronic Data Capture
STROBE	Strengthening the Reporting of Observational Studies in Epidemiology
SRQR	Standards for Reporting Qualitative Research
GRAMMS	Good Reporting of A Mixed Methods Study
HC3	Heteroskedasticity-consistent type 3 standard errors
CI	Confidence interval
SD	Standard deviation
IQR	Interquartile range

## References

[1] McLaney E., Morassaei S., Hughes L., Davies R., Campbell M., Di Prospero L. (2022). A Framework for Interprofessional Team Collaboration in a Hospital Setting: Advancing Team Competencies and Behaviours. Healthc. Manag. Forum.

[2] Wei H., Horns P., Sears S.F., Huang K., Smith C.M., Wei T.L. (2022). A Systematic Meta-Review of Systematic Reviews about Interprofessional Collaboration: Facilitators, Barriers, and Outcomes. J. Interprof. Care.

[3] Kaiser L., Conrad S., Neugebauer E.A.M., Pietsch B., Pieper D. (2022). Interprofessional Collaboration and Patient-Reported Outcomes in Inpatient Care: A Systematic Review. Syst. Rev..

[4] Gilbert J.H.V., Yan J., Hoffman S.J. (2010). A WHO Report: Framework for Action on Interprofessional Education and Collaborative Practice. J. Allied Health.

[5] McNeill D., Brown P. (2024). New IPEC Competencies, the State of the Science, and a Focus on Equity. N. C. Med. J..

[6] Patel H., Perry S., Badu E., Mwangi F., Onifade O., Mazurskyy A., Walters J., Tavener M., Noble D., Chidarikire S. (2025). A Scoping Review of Interprofessional Education in Healthcare: Evaluating Competency Development, Educational Outcomes and Challenges. BMC Med. Educ..

[7] Saragih I.D., Hsiao C.-T., Fann W.-C., Hsu C.-M., Saragih I.S., Lee B.-O. (2024). Impacts of Interprofessional Education on Collaborative Practice of Healthcare Professionals: A Systematic Review and Meta-Analysis. Nurse Educ. Today.

[8] El Nsouli D., Nelson D., Nsouli L., Curtis F., Ahmed S.I., McGonagle I., Kane R., Ahmadi K. (2023). The Application of Kirkpatrick’s Evaluation Model in the Assessment of Interprofessional Simulation Activities Involving Pharmacy Students: A Systematic Review. Am. J. Pharm. Educ..

[9] Oudbier J., Verheijck E., van Diermen D., Tams J., Bramer J., Spaai G. (2024). Enhancing the Effectiveness of Interprofessional Education in Health Science Education: A State-of-the-Art Review. BMC Med. Educ..

[10] Allvin R., Thompson C., Edelbring S. (2023). Variations in Measurement of Interprofessional Core Competencies: A Systematic Review of Self-Report Instruments in Undergraduate Health Professions Education. J. Interprof. Care.

[11] Maddock B., Dārziņš P., Kent F. (2022). Realist Review of Interprofessional Education for Health Care Students: What Works for Whom and Why. J. Interprof. Care.

[12] Kaas-Mason S., Langlois S., Bartlett S., Friesen F., Ng S., Bellicoso D., Rowland P. (2024). A Critical Interpretive Synthesis of Interprofessional Education Interventions. J. Interprof. Care.

[13] Bogossian F., New K., George K., Barr N., Dodd N., Hamilton A.L., Nash G., Masters N., Pelly F., Reid C. (2022). The Implementation of Interprofessional Education: A Scoping Review. Adv. Health Sci. Educ. Theory Pract..

[14] Farrukh K., Zehra F., Shahid F., Mehr Y. (2024). Health Sciences Faculty Attitude and Readiness Towards Simulation-Based Inter-Professional Education. J. Coll. Physicians Surg. Pak..

[15] Posey K., Prol L. (2024). Nurse Practitioner Faculty Attitudes about Interprofessional Education. J. Am. Assoc. Nurse Pract..

[16] Etherington C., Burns J.K., Kitto S., Brehaut J.C., Britton M., Singh S., Boet S. (2021). Barriers and Enablers to Effective Interprofessional Teamwork in the Operating Room: A Qualitative Study Using the Theoretical Domains Framework. PLoS ONE.

[17] Spada F., Caruso R., De Maria M., Karma E., Oseku A., Pata X., Prendi E., Rocco G., Notarnicola I., Stievano A. (2022). Italian Translation and Validation of the Readiness for Interprofessional Learning Scale (RIPLS) in an Undergraduate Healthcare Student Context. Healthcare.

[18] Spada F., Caruso R., Notarnicola I., De Maria M., Duka B., Arapi A., Prendi E., Rocco G., Stievano A. (2023). Describing the Readiness for Interprofessional Education among University Students Attending Healthcare Programs: Insights from a Monocentric Cross-Sectional Study. Acta Biomed..

[19] Spada F., Caruso R., Notarnicola I., Belloni S., De Maria M., Magon A., Conte G., Prendi E., Pata X., Duka B. (2025). Analyzing Readiness for Interprofessional Education among Health Program Students Using Hierarchical Clustering. J. Interprof. Care.

[20] Orchard C.A., King G.A., Khalili H., Bezzina M.B. (2012). Assessment of Interprofessional Team Collaboration Scale (AITCS): Development and Testing of the Instrument. J. Contin. Educ. Health Prof..

[21] Orchard C., Pederson L.L., Read E., Mahler C., Laschinger H. (2018). Assessment of Interprofessional Team Collaboration Scale (AITCS): Further Testing and Instrument Revision. J. Contin. Educ. Health Prof..

[22] Caruso R., Magon A., Dellafiore F., Griffini S., Milani L., Stievano A., Orchard C.A. (2018). Italian Version of the Assessment of Interprofessional Team Collaboration Scale II (I-AITCS II): A Multiphase Study of Validity and Reliability amongst Healthcare Providers. Med. Lav..

[23] Boonmak P., Saensom D., Tangpukdee J., Ruaisungnoen W., Chanthapasa K., Chaibunruang A., Kraiklang R., Limmonthol S., Phimphasak C., Boonmak P. (2024). Perceptions and Influencing Factors of Interprofessional Collaboration in Final-Year Health Science Students. J. Interprof. Care.

[24] Pun B.T., Jun J., Tan A., Byrum D., Mion L., Vasilevskis E.E., Ely E.W., Balas M. (2022). Interprofessional Team Collaboration and Work Environment Health in 68 US Intensive Care Units. Am. J. Crit. Care.

[25] Tashakkori A., Teddlie C., Johnson B. (2015). Mixed Methods. International Encyclopedia of the Social & Behavioral Sciences.

[26] von Elm E., Altman D.G., Egger M., Pocock S.J., Gøtzsche P.C., Vandenbroucke J.P. (2008). The Strengthening the Reporting of Observational Studies in Epidemiology (STROBE) Statement: Guidelines for Reporting Observational Studies. J. Clin. Epidemiol..

[27] O’Brien B.C., Harris I.B., Beckman T.J., Reed D.A., Cook D.A. (2014). Standards for Reporting Qualitative Research: A Synthesis of Recommendations. Acad. Med..

[28] O’cathain A., Murphy E., Nicholl J. (2008). The Quality of Mixed Methods Studies in Health Services Research. J. Health Serv. Res. Policy.

[29] Committee on Measuring the Impact of Interprofessional Education on Collaborative Practice and Patient Outcomes; Board on Global Health. Institute of Medicine. Measuring the Impact of Interprofessional Education on Collaborative Practice and Patient Outcomes. https://www.ncbi.nlm.nih.gov/books/NBK338356/.

[30] Faul F., Erdfelder E., Buchner A., Lang A.-G. (2009). Statistical Power Analyses Using G*Power 3.1: Tests for Correlation and Regression Analyses. Behav. Res. Methods.

[31] Kalpourtzi N., Carpenter J.R., Touloumi G. (2024). Handling Missing Values in Surveys With Complex Study Design: A Simulation Study. J. Surv. Stat. Methodol..

[32] Harris P.A., Taylor R., Thielke R., Payne J., Gonzalez N., Conde J.G. (2009). Research Electronic Data Capture (REDCap)—A Metadata-Driven Methodology and Workflow Process for Providing Translational Research Informatics Support. J. Biomed. Inform..

[33] Matthews R.A., Pineault L., Hong Y.-H. (2022). Normalizing the Use of Single-Item Measures: Validation of the Single-Item Compendium for Organizational Psychology. J. Bus. Psychol..

[34] Curran-Everett D. (2018). Explorations in Statistics: The Log Transformation. Adv. Physiol. Educ..

[35] Caruso R. Deidentified Quantitative Dataset on Interprofessional Education and Perceived Interprofessional Collaboration among Health Professions Faculty and Educators. https://figshare.com/articles/dataset/Deidentified_Quantitative_Dataset_on_Interprofessional_Education_and_Perceived_Interprofessional_Collaboration_among_Health_Professions_Faculty_and_Educators/33106577.

[36] Park C.G. (2024). Implementing Alternative Estimation Methods to Evaluate the Reliability of Likert-Scale Instruments. Womens Health Nurs..

[37] Chen F.F. (2007). Sensitivity of Goodness of Fit Indexes to Lack of Measurement Invariance. Struct. Equ. Model. A Multidiscip. J..

[38] Hsieh H.-F., Shannon S.E. (2005). Three Approaches to Qualitative Content Analysis. Qual. Health Res..

[39] Assarroudi A., Heshmati Nabavi F., Armat M.R., Ebadi A., Vaismoradi M. (2018). Directed Qualitative Content Analysis: The Description and Elaboration of Its Underpinning Methods and Data Analysis Process. J. Res. Nurs..

[40] Graneheim U.H., Lindgren B.-M., Lundman B. (2017). Methodological Challenges in Qualitative Content Analysis: A Discussion Paper. Nurse Educ. Today.

[41] Fetters M.D., Curry L.A., Creswell J.W. (2013). Achieving Integration in Mixed Methods Designs—Principles and Practices. Health Serv. Res..

[42] Guetterman T.C., Fetters M.D., Creswell J.W. (2015). Integrating Quantitative and Qualitative Results in Health Science Mixed Methods Research Through Joint Displays. Ann. Fam. Med..

[43] Brown G.T.L., Zhao A. (2023). In Defence of Psychometric Measurement: A Systematic Review of Contemporary Self-Report Feedback Inventories. Educ. Psychol..

[44] Weller J.M., Mahajan R., Fahey-Williams K., Webster C.S. (2024). Teamwork Matters: Team Situation Awareness to Build High-Performing Healthcare Teams, a Narrative Review. Br. J. Anaesth..

[45] Whitney K., Tucker P. (2026). Interprofessional Leadership. Nurse Lead..

[46] Simons M., Goossensen A., Nies H. (2022). Interventions Fostering Interdisciplinary and Inter-Organizational Collaboration in Health and Social Care; an Integrative Literature Review. J. Interprof. Educ. Pract..

[47] Zettna N., Yam C., Kunzelmann A., Forner V.W., Dey S., Askovic M., Johnson A., Nguyen H., Jolly A., Parker S.K. (2025). Crystal Clear: How Leaders and Coworkers Together Shape Role Clarity and Well-being for Employees in Social Care. Hum. Resour. Manag..

[48] Yousuf N., Atta K., Bhargava A., Gulzar S., Sandars J., Zaidi S., Zaidi A.N. (2026). Interprofessional Education of the Future. Modern Medical Education.

[49] Kvarnström M., Edelbring S., Lindh Falk A. (2025). The What and Why of Interprofessional Collaboration According to First-Year Health Professions Students: A Thematic Analysis of Written Reflections. BMC Med. Educ..

[50] Tashakkori A., Johnson R.B., Teddlie C. (2021). Foundations of Mixed Methods Research.

[51] Alsharari T., Khattak O., Agarwal A., Chaudhary F.A., Suhail N., Begum G.S., Subramaniam G., Albagami H., Felemban M.F., Shawli H.T. (2025). Health Professional Students’ Perceptions and Preparedness for Interprofessional Education: A Multicentric Analysis across Five Countries. Front. Med..

[52] Beebe T., Hazelwood A., Franklin E. (2025). “IPE Is Key”: Qualitative Findings of a Foundational Interprofessional Education Readiness Activity with Nursing and Health Information Management Students. Adv. Health Inf. Sci. Pract..

[53] Finch H., Michalos A.C. (2014). Measurement Invariance. Encyclopedia of Quality of Life and Well-Being Research.

[54] Hermasari B.K., Rahayu G.R., Pamungkasari E.P. (2026). Longitudinal Interprofessional Education in Health Profession Education: A Scoping Review. KJME.

[55] Ronchi S., Caruso R., Fabrizi D., Luciani M., Alfes C.M., Colley N., Magon A., Conte G., Valli A., Terzoni S. (2026). Virtual Reality in Undergraduate and Postgraduate Nursing Education:A Scoping Review Integrating Data Mining for Topic Discovery. Teach. Learn. Nurs..

[56] Lipnevich A.A., Mattern K., Feddock C. (2026). Formative Assessment and Feedback in Medical Education: A Practical Guide: AMEE Guide No. 189. Med. Teach..

[57] Octavia D.R., Hermansyah A., Nita Y., Asmani F., Abdullah I. (2026). The Feasibility of Implementing Interprofessional Collaboration-Based Telecare Services for Patients with Tuberculosis: A Mixed Methods Study from Hospital Insight. Interdiscip. Perspect. Infect. Dis..

