# Supplementary material for: Interprofessional Education Experience, Self-Rated Knowledge, and Perceived Interprofessional Collaboration Among Health Professions Faculty and Educators: A Cross-Sectional Mixed-Methods Study in Albania and Italy
Source: Healthcare (Basel). 2026 Aug 20;14(16):2649. doi: 10.3390/healthcare14162649 (PMC13512561; doi:10.3390/healthcare14162649)
Supplement: Supplementary file 1 [file healthcare-14-02649-s001.zip › Supplementary File S2 STROBE Checklist.pdf]

Supplementary File S2 - Completed STROBE Checklist

Interprofessional education and perceived interprofessional collaboration among health professions faculty and educators in Albanian and Italian working contexts

Completion note. Locations refer to the current numbered manuscript sections rather than final typeset pages. Items marked Partial or Pending require author confirmation before submission.

Guideline source: von Elm et al. (2008), J Clin Epidemiol, 61(4), 344-349. doi:10.1016/j.jclinepi.2007.11.008

Item	Topic	Reporting recommendation	Location and completion status
1a	Title and abstract	Identify the study design using a commonly used term in the title or abstract.	Reported - Abstract, Methods 2.1.
1b	Title and abstract	Provide an informative and balanced abstract summary of methods and findings.	Reported - Abstract.
2	Background/rationale	Explain the scientific background and rationale.	Reported - Introduction.
3	Objectives	State specific objectives and any prespecified hypotheses.	Reported - Study aim; Methods 2.1.
4	Study design	Present the key design elements early.	Reported - Methods 2.1.
5	Setting	Describe setting, locations, and relevant recruitment and data-collection dates.	Reported - Methods 2.2.
6	Participants	Give eligibility criteria and participant sources and selection methods.	Reported - Methods 2.2.
7	Variables	Define outcomes, exposures, predictors, confounders, and effect modifiers.	Reported - Methods 2.3; Table S1.
8	Data sources/measurement	Describe data sources and measurement methods for each variable and comparability across groups.	Reported - Methods 2.3.1-2.3.4; Table S1.
9	Bias	Describe efforts to address potential sources of bias.	Reported - Methods 2.2 and 2.4; Discussion 4.8.
10	Study size	Explain how the study size was determined.	Reported - Methods 2.2.
11	Quantitative variables	Explain how quantitative variables and any groupings were handled.	Reported - Methods 2.4.1-2.4.6; Table S1.
12a	Statistical methods	Describe statistical methods, including control of confounding.	Reported - Methods 2.4.2-2.4.5.
12b	Statistical methods	Describe subgroup and interaction analyses.	Reported - Methods 2.4.5.
12c	Statistical methods	Explain how missing data were addressed.	Reported - Methods 2.4.1.
12d	Statistical methods	Describe methods accounting for the sampling strategy, if applicable.	Not applicable - convenience recruitment from accessible institutional lists; no survey weighting.
12e	Statistical methods	Describe sensitivity analyses.	Reported - Methods 2.4.6; Tables S7a-S7g.
13a	Participants	Report numbers of individuals at each study stage.	Reported - Results 3.1; Figure S1.
13b	Participants	Give reasons for non-participation at each stage.	Results 3.1 and Figure S1 report the number of questionnaires not returned. Characteristics and reasons for non-participation were not collected.
13c	Participants	Consider a flow diagram.	Reported - Figure S1.
14a	Descriptive data	Report participant characteristics, exposures, and potential confounders.	Reported - Results 3.1; Table 1.
14b	Descriptive data	Report missing observations for each variable of interest.	Reported - Table S2; Figure S2.
15	Outcome data	Report outcome summary measures.	Reported - Results 3.2; Table 2.
16a	Main results	Report unadjusted and adjusted estimates with precision and state the adjustment set.	Reported - Results 3.5; Table 3; Table S6a.
16b	Main results	Report category boundaries when continuous variables were categorised.	Reported - Methods 2.4.1; Table S1.
16c	Main results	Translate relative risks into absolute risks when relevant.	Not applicable - continuous cross-sectional outcome models; no relative-risk estimates.
17	Other analyses	Report subgroup, interaction, and sensitivity analyses.	Reported - Results 3.5-3.6; Tables S6d-S7g.
18	Key results	Summarise key findings with reference to study objectives.	Reported - Discussion 4.1.
19	Limitations	Discuss study limitations and potential bias, including direction and magnitude where possible.	Reported - Discussion 4.8.

Item	Topic	Reporting recommendation	Location and completion status
20	Interpretation	Provide a cautious overall interpretation considering objectives, limitations, multiplicity, and other evidence.	Reported - Discussion 4.1-4.7.
21	Generalisability	Discuss external validity and generalisability.	Reported - Discussion 4.5 and 4.8.
22	Funding	State the funding source and the role of funders.	Done.
