# Supplementary material for: Interprofessional Education Experience, Self-Rated Knowledge, and Perceived Interprofessional Collaboration Among Health Professions Faculty and Educators: A Cross-Sectional Mixed-Methods Study in Albania and Italy
Source: Healthcare (Basel). 2026 Aug 20;14(16):2649. doi: 10.3390/healthcare14162649 (PMC13512561; doi:10.3390/healthcare14162649)
Supplement: Supplementary file 1 [file healthcare-14-02649-s001.zip › Supplementary File S3 SRQR Checklist.pdf]

Supplementary File S3 - Completed SRQR Checklist

Interprofessional education and perceived interprofessional collaboration among health professions faculty and educators in Albanian and Italian working contexts

Completion note. Locations refer to the current numbered manuscript sections rather than final typeset pages. Items marked Partial or Pending require author confirmation before submission.

Guideline source: O'Brien et al. (2014), Academic Medicine, 89(9), 1245-1251. doi:10.1097/ACM.0000000000000388

Item	Topic	Reporting recommendation	Location and completion status
S1	Title	Identify the qualitative nature, approach, or qualitative data-collection method in the title where appropriate.	Reported - mixed-methods design is explicit in Abstract and Methods 2.1.
S2	Abstract	Summarise background, purpose, methods, results, and conclusions.	Reported - Abstract.
S3	Problem formulation	Describe the problem, its significance, and relevant theory or empirical work.	Reported - Introduction.
S4	Purpose/research question	State the qualitative purpose or research question.	Reported - Study aim; Methods 2.1 and 2.5.2.
S5	Qualitative approach/paradigm	Name and justify the qualitative approach and, where relevant, the guiding paradigm or theory.	Reported - Methods
S6	Researcher characteristics/reflexivity	Report researcher characteristics, relationships, assumptions, or experience that may influence the study.	Reported - reflexive procedures are described in Methods 2.5.3; Supplementary File S1.
S7	Context	Describe the setting and contextual factors relevant to findings.	Reported - Methods 2.2 and 2.5.1; Discussion 4.5 and 4.8.
S8	Sampling strategy	Explain participant selection and rationale.	Reported - Methods 2.5.1; Table S8.
S9	Ethical issues	Describe ethics approval, consent, and confidentiality.	Reported - Methods 2.7.
S10	Data collection methods	Describe the data collected, collection procedures, dates, iterative changes, and relevant triangulation.	Reported - Methods 2.3.1, 2.3.5, and 2.5.1.
S11	Data collection instruments/technologies	Describe instruments and technologies used and provide them where appropriate.	Reported - Methods 2.3.1 and 2.3.5; Table S1.
S12	Units of study	Describe the number and characteristics of participants, documents, or events included.	Reported - Methods 2.5.1; Results 3.1 and 3.7; Table S8.
S13	Data processing	Describe preparation and management of data before and during analysis.	Reported - Methods 2.5.1-2.5.3; Supplementary File S1.
S14	Data analysis	Describe the analytic process in sufficient detail.	Reported - Methods 2.5.2; Supplementary File S1.
S15	Trustworthiness	Describe techniques used to enhance credibility and trustworthiness.	Reported - Methods 2.5.3; Table S9; Supplementary File S1.
S16	Synthesis and interpretation	Present the main qualitative findings and interpretations.	Reported - Results 3.7 and 3.8; Table 4.
S17	Links to empirical data	Support analytic claims with empirical data or examples.	Reported - Results 3.7; qualitative exemplars in Table 4 and Supplementary File S1.
S18	Integration with prior work and implications	Relate findings to prior research and discuss implications, transferability, and contribution.	Reported - Discussion 4.2-4.7.
S19	Limitations	Discuss limitations of the qualitative work.	Reported - Discussion 4.8.
S20	Conflicts of interest	Disclose potential sources of influence or perceived influence.	Done.
S21	Funding	Disclose funding sources and funder roles.	Done
