# Supplementary material for: Interprofessional Education Experience, Self-Rated Knowledge, and Perceived Interprofessional Collaboration Among Health Professions Faculty and Educators: A Cross-Sectional Mixed-Methods Study in Albania and Italy
Source: Healthcare (Basel). 2026 Aug 20;14(16):2649. doi: 10.3390/healthcare14162649 (PMC13512561; doi:10.3390/healthcare14162649)
Supplement: Supplementary file 1 [file healthcare-14-02649-s001.zip › Supplementary File S4 GRAMMS Checklist.pdf]

Supplementary File S4 - Completed GRAMMS Checklist

Interprofessional education and perceived interprofessional collaboration among health professions faculty and educators in Albanian and Italian working contexts

Completion note. Locations refer to the current numbered manuscript sections rather than final typeset pages. Items marked Partial or Pending require author confirmation before submission.

Guideline source: O'Cathain, Murphy, and Nicholl (2008), J Health Serv Res Policy, 13(2), 92-98. doi:10.1258/jhsrp.2007.007074

Item	Topic	Reporting recommendation	Location and completion status
1	Rationale	Justify why mixed methods were required for the research question.	Reported - Introduction and Study aim; Methods 2.1.
2	Design	Describe the mixed-methods purpose, priority, and sequence.	Reported - Methods 2.1: quantitative-dominant design; concurrent collection with sequential analysis (QUAN -> qual).
3	Components	Describe sampling, data collection, and analysis for each quantitative and qualitative component.	Reported - Methods 2.2-2.5.
4	Integration	Describe where and how integration occurred and who participated in it.	Reported - design, analysis, and reporting integration are described in Methods 2.6 and Results 3.8; identify the team members or roles who performed integration.
5	Limitations across methods	Describe limitations of one method associated with the presence of the other.	Reported - Discussion 4.8: aggregate rather than respondent-level integration, voluntary nested qualitative subsample, and geographical asymmetry.
6	Insights from mixing	Describe insights gained specifically through mixing or integrating methods.	Reported - Results 3.8 and Table 4; Discussion 4.1-4.7.
