# Supplementary material for: Interprofessional Education Experience, Self-Rated Knowledge, and Perceived Interprofessional Collaboration Among Health Professions Faculty and Educators: A Cross-Sectional Mixed-Methods Study in Albania and Italy
Source: Healthcare (Basel). 2026 Aug 20;14(16):2649. doi: 10.3390/healthcare14162649 (PMC13512561; doi:10.3390/healthcare14162649)

SUPPLEMENTARY TABLES AND FIGURES

Interprofessional education and perceived interprofessional collaboration among health professions faculty and educators in Albanian and Italian working contexts

CONTENTS

Table S1. Variable definitions, response formats, scoring rules, and analytical roles.
Table S2. Data-quality and missingness summary.
Table S3. AITCS-II item-level statistics.
Table S4a. Preliminary analysis: internal consistency.
Table S4b. Preliminary internal structural validity: confirmatory factor-analysis fit.
Table S4c. Preliminary internal structural validity: standardised CFA factor loadings.
Table S4d. Pragmatic continuous-item measurement-invariance sensitivity.
Table S4e. CFA diagnostics: modification indices of at least 10.
Table S4f. CFA diagnostics: residual correlations with an absolute value of at least 0.05.
Table S5. Spearman correlation matrix.
Table S6a. Regression analyses: complete coefficient estimates.
Table S6b. Regression analyses: collinearity diagnostics.
Table S6c. Regression analyses: diagnostic statistics.
Table S6d. Exploratory working-context interaction tests.
Table S6e. Working-context-specific adjusted estimates.
Table S7a. Sensitivity analysis: adjusted domain-specific AITCS-II models.
Table S7b. Sensitivity analysis: complete-item scoring.
Table S7c. Sensitivity analysis: non-linearity in self-rated IPE knowledge.
Table S7d. Sensitivity analysis: median regression for ancillary concordance between self-reported IPE indicators.
Table S7e. Ancillary two-part concordance analysis between self-reported IPE indicators.
Table S7f. Sensitivity analysis: survey-source adjustment of the focal AITCS-II associations.
Table S7g. Sensitivity analysis: working-country coefficient with and without survey-source adjustment.
Table S8. Qualitative subsample and corpus characteristics.
Table S9. Qualitative analytical rigour, expert appraisal, and coding agreement.
Table S10. Complete qualitative category frequencies.
Figure S1. Flow of participants into the descriptive, measurement, regression, and nested qualitative analyses.
Figure S2. Variable-level missing-data profile.
Figure S3. Standardised factor loadings for the correlated three-factor AITCS-II model.
Figure S4. Spearman correlation heatmap for the main continuous variables.
Figure S5. Diagnostic plots for the final AITCS-II interaction model.

Terminology. AITCS-II is used for pooled analyses. I-AITCS II is reserved for the validated Italian-language version. All analyses are preliminary or exploratory where stated in the captions and notes.

Table S1. Variable definitions, response formats, scoring rules, and analytical roles.

Measure	Response format	Scoring	Analytical role
Prior IPE experience	No/yes	Binary indicator	Focal exposure
Self-rated IPE knowledge	0-100 self-rating	Per 10-point increase in regression models	Focal independent indicator
AITCS-II Partnership	8 items; 1 (never) to 5 (always)	Mean if at least 7 of 8 items completed	Descriptive outcome and domain sensitivity
AITCS-II Cooperation	8 items; 1 (never) to 5 (always)	Mean if at least 7 of 8 items completed	Descriptive outcome and domain sensitivity
AITCS-II Coordination	7 items; 1 (never) to 5 (always)	Mean if at least 6 of 7 items completed	Descriptive outcome and domain sensitivity
AITCS-II overall score	23 items; 1 (never) to 5 (always)	Mean if at least 19 of 23 items completed	Primary continuous outcome
Country of work	Albania/Italy/unresolved	Current working context; Italy combined across technical containers	Covariate and exploratory effect modifier
Profession	Physician/nurse/other specified/other or unspecified	Collapsed four-level factor	Covariate
Work experience	Years	Per 10-year increase	Covariate
Annual teaching hours	Hours per year	log(1+x) transformation	Covariate
Academic role	Junior/senior	Binary factor	Covariate
Open-ended question OA1	Optional written response	Manifest-content multiple coding, maximum three categories	Qualitative strategies
Open-ended question OA2	Optional written response	Manifest-content multiple coding, maximum three categories	Qualitative barriers
Open-ended question OA3	Optional written response	Exclusive ordinal intensity code plus up to three rationale codes	Qualitative importance and rationale

Note. Definitions refer to the pooled AITCS-II terminology used in the manuscript.

Table S2. Data-quality and missingness summary.

variable	missing_n	missing_pct
oa2	16	3.6
ore_insegn_anno	14	3.1
oa1	12	2.7
oa3	11	2.5
c3	7	1.6
co4	5	1.1
p2	5	1.1
p4	5	1.1
p5	5	1.1
titolo_studio	5	1.1
anni_esperienza	4	0.9
c4	4	0.9
eta	4	0.9
p3	4	0.9
p7	4	0.9
professione	4	0.9
c2	3	0.7
c6	3	0.7
co5	3	0.7
co6	3	0.7
p6	3	0.7
co3	2	0.5
conosc_ipe	2	0.5
p1	2	0.5
p8	2	0.5
c5	1	0.2
c7	1	0.2
co1	1	0.2
co2	1	0.2
co7	1	0.2
c1	0	0.0
c8	0	0.0
consenso	0	0.0
contesto_originale	0	0.0
esp_ipe	0	0.0
id_cartaceo	0	0.0
ruolo_acc	0	0.0

Note. Percentages use the full quantitative sample (N = 445) unless otherwise indicated.

Table S3. AITCS-II item-level statistics.

item	domain	n	missing_n	missing_pct	mean	sd	median	floor_pct	ceiling_pct	corrected_item_total_r
P1	Partnership	443	2	0.4	3.756	1.147	4.000	5.6	31.6	0.645
P2	Partnership	440	5	1.1	3.811	1.172	4.000	6.4	35.7	0.674
P3	Partnership	441	4	0.9	3.812	1.159	4.000	4.8	36.1	0.682
P4	Partnership	440	5	1.1	3.432	1.230	3.500	9.1	23.6	0.669
P5	Partnership	440	5	1.1	3.777	1.161	4.000	5.9	33.2	0.705
P6	Partnership	442	3	0.7	3.633	1.190	4.000	5.4	28.5	0.679
P7	Partnership	441	4	0.9	3.816	1.142	4.000	4.8	34.7	0.684
P8	Partnership	443	2	0.4	3.858	1.128	4.000	4.7	35.9	0.671
C1	Cooperation	445	0	0.0	3.874	0.921	4.000	1.6	27.2	0.465
C2	Cooperation	442	3	0.7	4.328	0.749	4.000	0.2	48.0	0.411
C3	Cooperation	438	7	1.6	4.263	0.773	4.000	0.5	42.5	0.443
C4	Cooperation	441	4	0.9	4.098	0.805	4.000	0.7	33.3	0.522
C5	Cooperation	444	1	0.2	4.115	0.762	4.000	0.2	33.3	0.387
C6	Cooperation	442	3	0.7	4.183	0.771	4.000	0.5	37.8	0.475
C7	Cooperation	444	1	0.2	4.275	0.773	4.000	0.5	45.0	0.444
C8	Cooperation	445	0	0.0	4.445	0.671	5.000	0.2	53.9	0.479
CO1	Coordination	444	1	0.2	4.052	0.904	4.000	1.1	36.3	0.561
CO2	Coordination	444	1	0.2	4.074	0.892	4.000	0.7	36.9	0.575
CO3	Coordination	443	2	0.4	4.020	0.940	4.000	1.6	35.4	0.578
CO4	Coordination	440	5	1.1	4.068	0.893	4.000	1.1	35.9	0.596
CO5	Coordination	442	3	0.7	4.077	0.898	4.000	0.7	38.2	0.592
CO6	Coordination	442	3	0.7	3.713	1.042	4.000	3.2	26.0	0.603
CO7	Coordination	444	1	0.2	3.437	1.103	3.000	5.2	19.1	0.611

Note. Floor and ceiling percentages refer to response categories 1 and 5, respectively.

Table S4a. Preliminary analysis: internal consistency.

scale	n_items	n_complete	cronbach_alpha	standardized_alpha	average_interitem_r	ordinal_alpha	composite_reliability
Partnership	8	423	0.959	0.959	0.744	0.970	0.970
Cooperation	8	426	0.861	0.863	0.441	0.897	0.898
Coordination	7	429	0.895	0.896	0.552	0.921	0.922
Overall AITCS-II	23	394	0.928	0.927	0.356	0.941	

Note. Very high alpha and average inter-item correlations may indicate item redundancy.

Table S4b. Preliminary internal structural validity: confirmatory factor-analysis

model	n	chisq	df	p_value	cfi	tli	rmsea	rmsea_low	rmsea_high	srmr
Three-factor correlated CFA	445	306.35	227	<0.001	0.970	0.967	0.050	0.041	0.060	0.035

Note. A correlated three-factor model was evaluated for Partnership, Cooperation, and Coordination.

Table S4c. Preliminary internal structural validity: standardised CFA factor

factor	item	standardized_loading	se	z	p_value
Partnership	P1	0.862	0.016	54.99	<0.001
Partnership	P2	0.898	0.012	75.02	<0.001
Partnership	P3	0.906	0.011	80.68	<0.001
Partnership	P4	0.885	0.013	70.46	<0.001
Partnership	P5	0.922	0.011	86.67	<0.001
Partnership	P6	0.907	0.011	81.87	<0.001
Partnership	P7	0.894	0.012	72.98	<0.001
Partnership	P8	0.895	0.012	73.42	<0.001
Cooperation	C1	0.688	0.031	22.17	<0.001
Cooperation	C2	0.678	0.036	18.69	<0.001
Cooperation	C3	0.739	0.030	24.21	<0.001
Cooperation	C4	0.795	0.024	32.92	<0.001
Cooperation	C5	0.679	0.032	21.07	<0.001
Cooperation	C6	0.724	0.031	23.60	<0.001
Cooperation	C7	0.730	0.029	25.02	<0.001
Cooperation	C8	0.757	0.032	23.51	<0.001
Coordination	CO1	0.783	0.025	31.77	<0.001
Coordination	CO2	0.779	0.024	32.87	<0.001
Coordination	CO3	0.769	0.024	32.26	<0.001
Coordination	CO4	0.825	0.020	40.73	<0.001
Coordination	CO5	0.805	0.023	35.15	<0.001
Coordination	CO6	0.796	0.022	36.40	<0.001
Coordination	CO7	0.791	0.022	35.62	<0.001

Note. Standardised estimates are reported.

Table S4d. Pragmatic continuous-item measurement-invariance sensitivity.

model	n	chisq	df	p_value	cfi	tli	rmsea	rmsea_low	rmsea_high	srmr	aic	bic	converged	delta_cfi	delta_rmsea
Configural (continuous-item MLR sensitivity)	442	580.04	454	<0.001	0.981	0.979	0.036	0.025	0.045	0.039	21159.11	21748.26	TRUE		
Metric (equal loadings; continuous-item MLR sensitivity)	442	613.78	474	<0.001	0.979	0.977	0.037	0.027	0.045	0.044	21150.91	21658.24	TRUE	-0.002	0.001
Scalar (equal loadings/intercepts; continuous-item MLR sensitivity)	442	623.10	494	<0.001	0.981	0.980	0.035	0.024	0.043	0.044	21119.01	21544.51	TRUE	0.002	-0.002

Note. This sensitivity analysis is supportive only and does not establish definitive ordinal invariance for the original five-category items.

Table S4e. CFA diagnostics: modification indices of at least 10.

lhs	operator	rhs	modification_index	expected_parameter_change	standardized_expected_change
Partnership	=~	c5	25.442	-0.160	-0.117
Coordination	=~	c5	17.454	-0.294	-0.216
Partnership	=~	c1	15.266	0.128	0.093

Note. No post hoc correlated residuals were added to the primary CFA.

Table S4f. CFA diagnostics: residual correlations with an absolute value of at least 0.05.

item_1	item_2	residual_correlation	absolute_residual	exceeds_0_10
p8	c1	0.109	0.109	TRUE
p6	c5	-0.100	0.100	FALSE
p8	co1	-0.097	0.097	FALSE
p1	c5	-0.090	0.090	FALSE
p4	c5	-0.084	0.084	FALSE
c4	c5	0.082	0.082	FALSE
c4	c6	-0.080	0.080	FALSE
p6	c2	-0.080	0.080	FALSE
co1	co2	0.071	0.071	FALSE
p2	c5	-0.070	0.070	FALSE
p3	c5	-0.070	0.070	FALSE
c7	co4	-0.070	0.070	FALSE
p7	c5	-0.069	0.069	FALSE
p5	c1	0.068	0.068	FALSE
c2	co5	0.067	0.067	FALSE
p7	c1	0.067	0.067	FALSE
p3	c1	0.066	0.066	FALSE
p5	c6	0.065	0.065	FALSE
p5	co7	0.064	0.064	FALSE
p7	c3	-0.064	0.064	FALSE
c6	co7	-0.064	0.064	FALSE
p7	c6	0.063	0.063	FALSE
p5	c5	-0.063	0.063	FALSE
p2	c1	0.062	0.062	FALSE
p6	c1	0.062	0.062	FALSE
p3	c7	-0.062	0.062	FALSE
c6	co5	0.061	0.061	FALSE
p8	c8	0.058	0.058	FALSE
co3	co6	-0.057	0.057	FALSE

Table S4f. CFA diagnostics: residual correlations with an absolute value of at least 0.05.

p3	c3	-0.056	0.056	FALSE
c2	c8	0.056	0.056	FALSE
c5	co4	-0.055	0.055	FALSE
c5	co5	0.055	0.055	FALSE
c1	co1	-0.054	0.054	FALSE
c3	co6	0.054	0.054	FALSE

item_1	item_2	residual_correlation	absolute_residual	exceeds_0_10
c1	co6	0.054	0.054	FALSE
c4	c8	-0.054	0.054	FALSE
p7	co3	0.054	0.054	FALSE
c3	co4	0.053	0.053	FALSE
c2	co2	0.053	0.053	FALSE
p3	co7	0.053	0.053	FALSE
p8	c4	0.053	0.053	FALSE
p2	co7	0.053	0.053	FALSE
co5	co6	-0.053	0.053	FALSE
p4	c1	0.052	0.052	FALSE
c3	co2	0.052	0.052	FALSE
c3	c8	-0.052	0.052	FALSE
p6	co1	-0.051	0.051	FALSE
p2	c3	-0.051	0.051	FALSE
c1	co7	0.051	0.051	FALSE
p2	c7	-0.050	0.050	FALSE
c1	c8	-0.050	0.050	FALSE

Note. Residual correlations are presented diagnostically; values above 0.10 are flagged in the source table.

Table S5. Spearman correlation matrix.

Variable	Age	Work experience	Annual teaching hours	IPE knowledge	Partnership	Cooperation	Coordination	Overall AITCS-II
Age	1.000	0.935	0.100	-0.043	-0.027	0.079	0.026	0.005
Work experience	0.935	1.000	0.105	-0.047	0.012	0.081	0.010	0.030
Annual teaching hours	0.100	0.105	1.000	-0.043	-0.046	-0.047	-0.012	-0.052
IPE knowledge	-0.043	-0.047	-0.043	1.000	0.171	0.178	0.324	0.276
Partnership	-0.027	0.012	-0.046	0.171	1.000	0.274	0.421	0.830
Cooperation	0.079	0.081	-0.047	0.178	0.274	1.000	0.575	0.660
Coordination	0.026	0.010	-0.012	0.324	0.421	0.575	1.000	0.785
Overall AITCS-II	0.005	0.030	-0.052	0.276	0.830	0.660	0.785	1.000

Note. Pairwise Spearman correlations are shown for the principal continuous variables.

Table S6a. Regression analyses: complete coefficient estimates.

term	estimate	std_error	statistic	p_value	conf_low	conf_high	model	analysis_role	n	r_squared	adj_r_squared
(Intercept)	18.054	1.332	13.55	<0.001	15.436	20.672	Unadjusted	Ancillary	417	0.324	0.323
ipe_experienceYes	28.910	2.067	13.99	<0.001	24.848	32.972	Unadjusted	Ancillary	417	0.324	0.323
(Intercept)	34.245	5.786	5.92	<0.001	22.872	45.618	Adjusted	Ancillary	417	0.349	0.336
ipe_experienceYes	29.490	2.072	14.23	<0.001	25.416	33.563	Adjusted	Ancillary	417	0.349	0.336
countryItaly	-0.359	2.716	-0.13	0.895	-5.699	4.980	Adjusted	Ancillary	417	0.349	0.336
profession_modelNurse	-7.244	4.242	-1.71	0.088	-15.582	1.095	Adjusted	Ancillary	417	0.349	0.336
profession_modelOther specified health profession	-1.776	4.473	-0.40	0.692	-10.569	7.018	Adjusted	Ancillary	417	0.349	0.336
profession_modelOther/unspecified	-4.952	2.401	-2.06	0.040	-9.672	-0.232	Adjusted	Ancillary	417	0.349	0.336
experience_years_10	-0.862	0.998	-0.86	0.388	-2.824	1.100	Adjusted	Ancillary	417	0.349	0.336
log_teaching_hours	-1.556	1.120	-1.39	0.165	-3.758	0.645	Adjusted	Ancillary	417	0.349	0.336
academic_roleSenior	-5.460	3.363	-1.62	0.105	-12.071	1.151	Adjusted	Ancillary	417	0.349	0.336
(Intercept)	3.773	0.043	86.78	<0.001	3.687	3.858	Experience unadjusted	Supporting	417	0.102	0.100
ipe_experienceYes	0.397	0.057	6.95	<0.001	0.284	0.509	Experience unadjusted	Supporting	417	0.102	0.100
(Intercept)	4.110	0.168	24.53	<0.001	3.781	4.439	Experience adjusted	Supporting	417	0.129	0.112
ipe_experienceYes	0.410	0.057	7.20	<0.001	0.298	0.522	Experience adjusted	Supporting	417	0.129	0.112

Table S6a. Regression analyses: complete coefficient estimates.

countryItaly	-0.113	0.079	-1.43	0.155	-0.268	0.043	Experience adjusted	Supporting	417	0.129	0.112
profession_modelNurse	0.086	0.087	0.99	0.325	-0.085	0.256	Experience adjusted	Supporting	417	0.129	0.112
profession_modelOther specified health profession	0.092	0.120	0.76	0.446	-0.145	0.329	Experience adjusted	Supporting	417	0.129	0.112
profession_modelOther/unspecified	-0.112	0.070	-1.60	0.111	-0.248	0.026	Experience adjusted	Supporting	417	0.129	0.112
experience_years_10	0.030	0.026	1.14	0.254	-0.022	0.082	Experience adjusted	Supporting	417	0.129	0.112
log_teaching_hours	-0.047	0.033	-1.40	0.162	-0.112	0.019	Experience adjusted	Supporting	417	0.129	0.112
academic_roleSenior	-0.120	0.085	-1.42	0.157	-0.286	0.046	Experience adjusted	Supporting	417	0.129	0.112
(Intercept)	3.948	0.174	22.64	<0.001	3.606	4.291	Knowledge adjusted	Supporting	417	0.095	0.078
knowledge_ipe_10	0.067	0.011	5.90	<0.001	0.045	0.089	Knowledge adjusted	Supporting	417	0.095	0.078
countryItaly	-0.097	0.080	-1.20	0.230	-0.254	0.061	Knowledge adjusted	Supporting	417	0.095	0.078
profession_modelNurse	0.129	0.101	1.28	0.202	-0.069	0.327	Knowledge adjusted	Supporting	417	0.095	0.078
profession_modelOther specified health profession	0.092	0.116	0.79	0.428	-0.136	0.320	Knowledge adjusted	Supporting	417	0.095	0.078
profession_modelOther/unspecified	-0.073	0.070	-1.04	0.298	-0.209	0.064	Knowledge adjusted	Supporting	417	0.095	0.078
experience_years_10	0.039	0.028	1.42	0.155	-0.015	0.093	Knowledge adjusted	Supporting	417	0.095	0.078
log_teaching_hours	-0.037	0.033	-1.11	0.268	-0.102	0.029	Knowledge adjusted	Supporting	417	0.095	0.078
academic_roleSenior	-0.065	0.086	-0.76	0.449	-0.234	0.104	Knowledge adjusted	Supporting	417	0.095	0.078
(Intercept)	4.007	0.173	23.12	<0.001	3.667	4.348	Joint adjusted	Primary	417	0.139	0.120

Table S6a. Regression analyses: complete coefficient estimates.

ipe_experienceYes	0.322	0.073	4.38	<0.001	0.177	0.466	Joint adjusted	Primary	417	0.139	0.120
knowledge_ipe_10	0.030	0.014	2.11	0.035	0.002	0.058	Joint adjusted	Primary	417	0.139	0.120
countryItaly	-0.111	0.079	-1.42	0.158	-0.266	0.043	Joint adjusted	Primary	417	0.139	0.120
profession_modelNurse	0.107	0.088	1.22	0.222	-0.065	0.280	Joint adjusted	Primary	417	0.139	0.120
profession_modelOther specified health profession	0.097	0.117	0.83	0.406	-0.133	0.327	Joint adjusted	Primary	417	0.139	0.120
profession_modelOther/unspecified	-0.097	0.069	-1.41	0.161	-0.232	0.039	Joint adjusted	Primary	417	0.139	0.120
experience_years_10	0.033	0.027	1.23	0.219	-0.019	0.085	Joint adjusted	Primary	417	0.139	0.120
log_teaching_hours	-0.042	0.033	-1.28	0.201	-0.106	0.022	Joint adjusted	Primary	417	0.139	0.120
academic_roleSenior	-0.104	0.085	-1.22	0.224	-0.271	0.064	Joint adjusted	Primary	417	0.139	0.120

term	estimate	std_error	statistic	p_value	conf_low	conf_high	model	analysis_role	n	r_squared	adj_r_squared
(Intercept)	31.491	6.480	4.86	<0.001	18.753	44.228	Context interaction	Ancillary	417	0.353	0.339
ipe_experienceYes	36.018	4.958	7.26	<0.001	26.271	45.766	Context interaction	Ancillary	417	0.353	0.339
countryItaly	3.147	3.449	0.91	0.362	-3.633	9.927	Context interaction	Ancillary	417	0.353	0.339
profession_modelNurse	-7.309	4.190	-1.74	0.082	-15.546	0.927	Context interaction	Ancillary	417	0.353	0.339
profession_modelOther specified health profession	-1.929	4.479	-0.43	0.667	-10.734	6.875	Context interaction	Ancillary	417	0.353	0.339
profession_modelOther/unspecified	-5.041	2.397	-2.10	0.036	-9.754	-0.329	Context interaction	Ancillary	417	0.353	0.339
experience_years_10	-0.902	1.000	-0.90	0.367	-2.867	1.063	Context interaction	Ancillary	417	0.353	0.339
log_teaching_hours	-1.461	1.136	-1.29	0.199	-3.694	0.772	Context interaction	Ancillary	417	0.353	0.339
academic_roleSenior	-5.747	3.390	-1.70	0.091	-12.411	0.916	Context interaction	Ancillary	417	0.353	0.339
ipe_experienceYes:countryItaly	-8.174	5.449	-1.50	0.134	-18.886	2.539	Context interaction	Ancillary	417	0.353	0.339
(Intercept)	4.021	0.186	21.57	<0.001	3.654	4.387	Experience x context	Exploratory	417	0.139	0.118
ipe_experienceYes	0.286	0.142	2.02	0.044	0.008	0.565	Experience x context	Exploratory	417	0.139	0.118
countryItaly	-0.130	0.118	-1.09	0.274	-0.363	0.103	Experience x context	Exploratory	417	0.139	0.118
knowledge_ipe_10	0.030	0.014	2.15	0.032	0.003	0.058	Experience x context	Exploratory	417	0.139	0.118
profession_modelNurse	0.108	0.088	1.23	0.220	-0.065	0.280	Experience x context	Exploratory	417	0.139	0.118
profession_modelOther specified health profession	0.098	0.118	0.83	0.406	-0.134	0.330	Experience x context	Exploratory	417	0.139	0.118

profession_modelOther/unspecified	-0.096	0.069	-1.38	0.167	-0.233	0.040	Experience x context	Exploratory	417	0.139	0.118
experience_years_10	0.033	0.027	1.24	0.217	-0.019	0.086	Experience x context	Exploratory	417	0.139	0.118
log_teaching_hours	-0.042	0.033	-1.29	0.197	-0.107	0.022	Experience x context	Exploratory	417	0.139	0.118
academic_roleSenior	-0.102	0.086	-1.19	0.234	-0.271	0.066	Experience x context	Exploratory	417	0.139	0.118
ipe_experienceYes:countryItaly	0.043	0.149	0.29	0.774	-0.250	0.336	Experience x context	Exploratory	417	0.139	0.118
(Intercept)	3.971	0.206	19.27	<0.001	3.566	4.377	Joint context interactions	Exploratory	417	0.141	0.117
ipe_experienceYes	0.216	0.158	1.36	0.174	-0.096	0.527	Joint context interactions	Exploratory	417	0.141	0.117
countryItaly	-0.084	0.141	-0.60	0.550	-0.361	0.193	Joint context interactions	Exploratory	417	0.141	0.117
knowledge_ipe_10	0.051	0.031	1.64	0.102	-0.010	0.111	Joint context interactions	Exploratory	417	0.141	0.117
profession_modelNurse	0.112	0.088	1.27	0.205	-0.061	0.285	Joint context interactions	Exploratory	417	0.141	0.117
profession_modelOther specified health profession	0.107	0.119	0.90	0.371	-0.127	0.341	Joint context interactions	Exploratory	417	0.141	0.117
profession_modelOther/unspecified	-0.092	0.069	-1.34	0.181	-0.228	0.043	Joint context interactions	Exploratory	417	0.141	0.117
experience_years_10	0.032	0.027	1.20	0.231	-0.021	0.085	Joint context interactions	Exploratory	417	0.141	0.117
log_teaching_hours	-0.040	0.033	-1.21	0.229	-0.104	0.025	Joint context interactions	Exploratory	417	0.141	0.117
academic_roleSenior	-0.098	0.085	-1.14	0.253	-0.265	0.070	Joint context interactions	Exploratory	417	0.141	0.117
ipe_experienceYes:countryItaly	0.132	0.176	0.75	0.453	-0.213	0.477	Joint context interactions	Exploratory	417	0.141	0.117
countryItaly:knowledge_ipe_10	-0.027	0.034	-0.79	0.428	-0.094	0.040	Joint context interactions	Exploratory	417	0.141	0.117

Note. Linear regression estimates use heteroskedasticity-consistent HC3 standard errors. Analysis role: Ancillary denotes the experience-knowledge concordance analysis; Supporting denotes single-predictor collaboration models; Primary denotes the joint adjusted collaboration model; and Exploratory denotes context-interaction extensions. Models with self-rated IPE knowledge as the outcome are retained as supplementary ancillary analyses of concordance between two related self-reported indicators. Because the knowledge score showed marked lower-bound concentration and heaping, the two-part model in Supplementary Table S7e was considered more appropriate for describing this relationship. The conventional linear models are presented for analytical transparency and sensitivity purposes only and do not assess construct validity, educational effectiveness, or causality.

Table S6b. Regression analyses: collinearity diagnostics.

term	gvif	df	adjusted_gvif	model
ipe_experience	1.034	3	1.006	Ancillary concordance interaction
country	1.034	3	1.006	Ancillary concordance interaction
profession_model	1.034	3	1.006	Ancillary concordance interaction
experience_years_10	1.311	1	1.145	Ancillary concordance interaction
log_teaching_hours	1.034	1	1.017	Ancillary concordance interaction
academic_role	1.334	1	1.155	Ancillary concordance interaction
ipe_experience	5.507	3	1.329	Exploratory collaboration interaction
country	1.099	5	1.009	Exploratory collaboration interaction
knowledge_ipe_10	4.309	3	1.276	Exploratory collaboration interaction
profession_model	1.062	3	1.010	Exploratory collaboration interaction
experience_years_10	1.317	1	1.147	Exploratory collaboration interaction
log_teaching_hours	1.047	1	1.023	Exploratory collaboration interaction
academic_role	1.350	1	1.162	Exploratory collaboration interaction

Note. Adjusted generalised variance-inflation factors are reported where applicable. Collinearity diagnostics for the ancillary concordance model are reported for analytical transparency and should not be interpreted as evidence regarding the validity of the single-item knowledge measure.

Table S6c. Regression analyses: diagnostic statistics.

model	n	residual_sd	breusch_pagan_p	reset_p	max_cooks_distance	influential_n_4over_n	model_matrix_rank	model_matrix_columns
Ancillary concordance interaction	417	20.625	0.286	0.714	0.037	28	10	10
Exploratory collaboration interaction	417	0.582	0.013	0.765	0.059	22	12	12

Note. HC3 inference was retained because it does not require homoskedastic residual variance. Diagnostic statistics for the conventional linear concordance model are supplementary because the distribution of self-rated knowledge was better represented by the two-part analysis in Supplementary Table S7e.

Table S6d. Exploratory working-context interaction tests.

analysis_role	test	df	p_value
Ancillary concordance	Prior IPE experience x working context; outcome: self-rated IPE knowledge	1	0.134
Exploratory collaboration	Prior IPE experience x working context	1	0.453
Exploratory collaboration	Self-rated IPE knowledge x working context	1	0.428
Exploratory collaboration	Joint working-context interactions	2	0.677

Note. Interaction analyses were exploratory and were not separately powered for cross-context equivalence claims. The first interaction test belongs to the ancillary concordance analysis and was not used to validate the knowledge item or support context-specific educational inference. The remaining tests are exploratory extensions of the primary collaboration model.

Table S6e. Working-context-specific adjusted estimates.

role	outcome	predictor	country	estimate	std_error	statistic	p_value	conf_low	conf_high	n	r_squared	adj_r_squared	interaction_p
Ancillary concordance	Self-rated IPE knowledge (0-100)	Prior IPE experience: Yes vs No	Albania	36.018	4.958	7.26	<0.001	26.271	45.766	417	0.353	0.339	0.134
Ancillary concordance	Self-rated IPE knowledge (0-100)	Prior IPE experience: Yes vs No	Italy	27.845	2.271	12.26	<0.001	23.380	32.309	417	0.353	0.339	0.134
Exploratory collaboration	Overall AITCS-II mean score (1-5)	Prior IPE experience: Yes vs No	Albania	0.216	0.158	1.36	0.174	-0.096	0.527	417	0.141	0.117	0.453
Exploratory collaboration	Overall AITCS-II mean score (1-5)	Prior IPE experience: Yes vs No	Italy	0.347	0.082	4.23	<0.001	0.186	0.509	417	0.141	0.117	0.453
Exploratory collaboration	Overall AITCS-II mean score (1-5)	IPE knowledge: per 10-point increase	Albania	0.051	0.031	1.64	0.102	-0.010	0.111	417	0.141	0.117	0.428
Exploratory collaboration	Overall AITCS-II mean score (1-5)	IPE knowledge: per 10-point increase	Italy	0.024	0.016	1.51	0.131	-0.007	0.054	417	0.141	0.117	0.428

Note. Context-specific estimates are derived from the corresponding interaction models and should be interpreted cautiously. Estimates for self-rated IPE knowledge are retained as ancillary descriptive analyses. Given the distribution of the knowledge score, the two-part model in Supplementary Table S7e remains the preferred analysis of concordance. None of these estimates provides evidence of construct validity, educational effectiveness, or equivalence between working contexts.

Table S7a. Sensitivity analysis: adjusted domain-specific AITCS-II models.

term	estimate	std_error	statistic	p_value	conf_low	conf_high	model	analysis_role	n	r_squared	adj_r_squared	domain	p_bh_across_domains
ipe_experienceYes	0.388	0.127	3.06	0.002	0.139	0.636	Domain model: Partnership	Collaboration sensitivity	416	0.069	0.048	Partnership	0.004
knowledge_ipe_10	0.025	0.024	1.06	0.290	-0.022	0.072	Domain model: Partnership	Collaboration sensitivity	416	0.069	0.048	Partnership	0.290
ipe_experienceYes	0.172	0.065	2.67	0.008	0.045	0.299	Domain model: Cooperation	Collaboration sensitivity	417	0.077	0.057	Cooperation	0.008
knowledge_ipe_10	0.020	0.013	1.51	0.131	-0.006	0.046	Domain model: Cooperation	Collaboration sensitivity	417	0.077	0.057	Cooperation	0.197
ipe_experienceYes	0.419	0.088	4.77	<0.001	0.246	0.592	Domain model: Coordination	Collaboration sensitivity	417	0.159	0.141	Coordination	<0.001
knowledge_ipe_10	0.047	0.018	2.60	0.010	0.011	0.083	Domain model: Coordination	Collaboration sensitivity	417	0.159	0.141	Coordination	0.029

Note. Benjamini-Hochberg adjusted p-values are reported across the three domains.

Table S7b. Sensitivity analysis: complete-item scoring.

term	estimate	std_error	statistic	p_value	conf_low	conf_high	model	analysis_role	n	r_squared	adj_r_squared
ipe_experienceYes	0.365	0.077	4.75	<0.001	0.214	0.517	Collaboration: complete-item score sensitivity	Collaboration sensitivity	368	0.150	0.129
knowledge_ipe_10	0.024	0.015	1.62	0.105	-0.005	0.053	Collaboration: complete-item score sensitivity	Collaboration sensitivity	368	0.150	0.129

Note. Only participants with complete AITCS-II item data were included.

Table S7c. Sensitivity analysis: non-linearity in self-rated IPE knowledge.

term	estimate	std_error	statistic	p_value	conf_low	conf_high	model	analysis_role	n	r_squared	adj_r_squared
ipe_experienceYes	0.316	0.075	4.23	<0.001	0.169	0.463	Collaboration: quadratic knowledge sensitivity	Collaboration sensitivity	417	0.140	0.119
knowledge_ipe_10	0.052	0.032	1.60	0.111	-0.012	0.115	Collaboration: quadratic knowledge sensitivity	Collaboration sensitivity	417	0.140	0.119
I(knowledge_ipe_10^2)	-0.003	0.003	-0.87	0.387	-0.009	0.004	Collaboration: quadratic knowledge sensitivity	Collaboration sensitivity	417	0.140	0.119

Note. The analysis assesses whether the linear knowledge specification was materially inadequate.

Table S7d. Sensitivity analysis: median regression for ancillary concordance between self-reported IPE indicators.

term	estimate	std_error	statistic	p_value	model	n
ipe_experienceYes	40.000	2.414	16.57	<0.001	Ancillary concordance: median-regression sensitivity	417

Note. This supplementary analysis describes expected concordance between self-reported prior IPE experience and single-item self-rated IPE knowledge. It does not assess construct or criterion validity and is not interpreted as an educational or causal effect.

Table S7e. Ancillary two-part concordance analysis between self-reported IPE indicators

part	outcome	effect_measure	estimate	conf_low	conf_high	p_value	n
Part 1	Probability of self-rated IPE knowledge >0	Odds ratio	18.904	8.346	42.816	<0.001	417
Part 2	Self-rated IPE knowledge among values >0	Adjusted mean difference	19.471	15.363	23.579	<0.001	323

Note. Part 1 models the probability of reporting any self-rated IPE knowledge; Part 2 models the reported knowledge level among participants with positive values. Both indicators were self-reported and partly conceptually overlapping. The estimates describe expected concordance and do not provide evidence of construct or criterion validity, diagnostic accuracy, educational effectiveness, or causality.

Table S7f. Sensitivity analysis: survey-source adjustment of the focal AITCS-II associations.

Analysis role	outcome	predictor	country	estimate	std_error	statistic	p_value	conf_low	conf_high	n	r_squared	adj_r_squared	interaction_p	adjustment
Collaboration sensitivity	Overall AITCS-II mean score (1-5)	Prior IPE experience: Yes vs No	Albania	0.216	0.158	1.36	0.174	-0.096	0.527	417	0.141	0.118	0.453	Country only (primary)
Collaboration sensitivity	Overall AITCS-II mean score (1-5)	Prior IPE experience: Yes vs No	Italy	0.347	0.082	4.23	<0.001	0.186	0.509	417	0.141	0.118	0.453	Country only (primary)
Collaboration sensitivity	Overall AITCS-II mean score (1-5)	IPE knowledge: per 10-point increase	Albania	0.051	0.031	1.64	0.102	-0.010	0.111	417	0.141	0.118	0.428	Country only (primary)
Collaboration sensitivity	Overall AITCS-II mean score (1-5)	IPE knowledge: per 10-point increase	Italy	0.024	0.016	1.52	0.131	-0.007	0.054	417	0.141	0.118	0.428	Country only (primary)
Collaboration sensitivity	Overall AITCS-II mean score (1-5)	Prior IPE experience: Yes vs No	Albania	0.215	0.159	1.36	0.175	-0.096	0.527	417	0.141	0.115	0.453	Country + survey source
Collaboration sensitivity	Overall AITCS-II mean score (1-5)	Prior IPE experience: Yes vs No	Italy	0.347	0.082	4.22	<0.001	0.186	0.509	417	0.141	0.115	0.453	Country + survey source
Collaboration sensitivity	Overall AITCS-II mean score (1-5)	IPE knowledge: per 10-point increase	Albania	0.051	0.031	1.64	0.102	-0.010	0.111	417	0.141	0.115	0.428	Country + survey source
Collaboration sensitivity	Overall AITCS-II mean score (1-5)	IPE knowledge: per 10-point increase	Italy	0.024	0.016	1.51	0.133	-0.007	0.054	417	0.141	0.115	0.428	Country + survey source

Note. Survey source denotes the technical questionnaire container and is not equivalent to working-country context.

Table S7g. Sensitivity analysis: working-country coefficient with and without survey-source adjustment.

term	estimate	std_error	statistic	p_value	conf_low	conf_high	model	analysis_role	n	r_squared	adj_r_squared
countryItaly	-0.111	0.079	-1.41	0.158	-0.266	0.043	Collaboration: additive model, country only	Collaboration sensitivity	417	0.139	0.120
countryItaly	-0.113	0.146	-0.77	0.443	-0.400	0.175	Collaboration: additive model, country + survey source	Collaboration sensitivity	417	0.139	0.118

Note. The source-adjusted country coefficient is imprecise because the second survey container included only Italian-context responses.

Table S8. Qualitative subsample and corpus characteristics.

Component	n	Denominator	Percentage
Participants with analysable qualitative text	114	445	25.6
Working context: Albania	86	114	75.4
Working context: Italy	25	114	21.9
Working context unresolved	3	114	2.6
OA1 strategies responses	114	114	100.0
OA2 barriers responses	113	114	99.1
OA3 importance responses	114	114	100.0

Note. Percentages describing working-context composition use the qualitative subsample (n = 114) as the denominator. Working-context-specific participation percentages use the corresponding quantitative working-context group as the denominator. The qualitative component was a nested voluntary-response subsample and was not geographically representative of the full sample. No survey source was excluded a priori; reasons for completion or non-completion of the optional open-ended section were not collected

Table S9. Qualitative analytical rigour, expert appraisal, and coding agreement.

Section	Metric	Value	Detail
Content validity	A priori categories appraised	49	Two expert appraisers
Content validity	I-CVI range	1.00-1.00	All categories met the two-appraiser criterion
Content validity	S-CVI/Ave	1	Mean item-level content validity
Content validity	S-CVI/UA	1	Universal agreement across categories
Inter-coder agreement	OA1	Exact agreement 93.0%; kappa 0.920; mean Jaccard 0.978	Binary category kappa range 0.796-1.000 across 15 estimable categories
Inter-coder agreement	OA2	Exact agreement 93.8%; kappa 0.933; mean Jaccard 0.982	Binary category kappa range 0.904-1.000 across 18 estimable categories
Inter-coder agreement	OA3	Exact agreement 93.9%; kappa 0.929; mean Jaccard 0.982	Binary category kappa range 0.940-1.000 across 11 estimable categories
Inter-coder agreement	OA3 intensity	Exact agreement 97.4%; linear weighted kappa 0.983; quadratic weighted kappa 0.994	Ordinal I0-I4 classification
Codebook stability	OA1 new codes in final 25%	0	Category stability criterion met
Codebook stability	OA2 new codes in final 25%	1	Category stability criterion not met; no claim of full saturation
Codebook stability	OA3 new codes in final 25%	0	Category stability criterion met
Audit trail	Documentation	Complete	Decision log, codebook versions, consensus notes, and analytic memos retained

Note. Agreement estimates complement, but do not replace, consensus review, reflexive procedures, and the audit trail.

Table S10. Complete qualitative category frequencies.

Question	Axis	Code	Category	n	Denominator	Percentage	Inductively added category
OA1	Manifest category	A1	Revisione curriculare e insegnamenti condivisi	38	114	33.3	No
OA1	Manifest category	A2	Apprendimento esperienziale in aula	16	114	14.0	No
OA1	Manifest category	A3	Tirocinio e pratica clinica interprofessionale	13	114	11.4	No
OA1	Manifest category	A4	Formazione e collaborazione del corpo docente	13	114	11.4	No
OA1	Manifest category	A5	Occasioni strutturate di incontro e confronto	18	114	15.8	No
OA1	Manifest category	A6	Progetti e ricerca collaborativa	5	114	4.4	No
OA1	Manifest category	A7	Leadership istituzionale e governance	9	114	7.9	No
OA1	Manifest category	A8	Cambiamento culturale e di mentalità	10	114	8.8	No
OA1	Manifest category	A9	Comunicazione e linguaggio comune	18	114	15.8	No
OA1	Manifest category	A10	Chiarimento di ruoli e competenze	8	114	7.0	No
OA1	Manifest category	A11	Risorse, tempo e strutture dedicate	3	114	2.6	No
OA1	Manifest category	A12	Tecnologie digitali e piattaforme	1	114	0.9	No
OA1	Manifest category	A13	Collocazione temporale nel percorso formativo	5	114	4.4	No
OA1	Manifest category	A14	Integrazione longitudinale e routinaria	7	114	6.1	Yes
OA1	Manifest category	A98	Contesto già adeguato	2	114	1.8	No
OA1	Manifest category	A99	Nessuna proposta/non pertinente	17	114	14.9	No
OA2	Manifest category	B1	Rigidità curriculare e assenza di curriculum condiviso	9	113	8.0	No
OA2	Manifest category	B2	Vincoli organizzativi e logistici	18	113	15.9	No
OA2	Manifest category	B3	Mancanza di tempo e sovraccarico	16	113	14.2	No
OA2	Manifest category	B4	Risorse economiche e umane insufficienti	17	113	15.0	No
OA2	Manifest category	B5	Assenza di supporto istituzionale/politiche	7	113	6.2	No
OA2	Manifest category	B6	Burocrazia e lentezza decisionale	2	113	1.8	No
OA2	Manifest category	B7	Cultura dei silos e resistenza al cambiamento	21	113	18.6	No

OA2	Manifest category	B8	Gerarchie e asimmetrie di status	4	113	3.5	No
OA2	Manifest category	B9	Scarsa conoscenza e consapevolezza IPE	7	113	6.2	No
OA2	Manifest category	B10	Formazione pedagogica dei docenti insufficiente	9	113	8.0	No
OA2	Manifest category	B11	Scarsa motivazione o interesse	15	113	13.3	No
OA2	Manifest category	B12	Comunicazione/coordinamento carenti tra strutture	13	113	11.5	No
OA2	Manifest category	B13	Barriere linguistiche, generazionali o preparazione	9	113	8.0	No
OA2	Manifest category	B14	Ambiguità di ruoli e confini di competenza	8	113	7.1	No
OA2	Manifest category	B15	Mancanza di occasioni strutturate	6	113	5.3	No
OA2	Manifest category	B16	Assenza di esperienza pregressa/modelli consolidati	5	113	4.4	No
OA2	Manifest category	B98	Nessun ostacolo percepito	5	113	4.4	No
OA2	Manifest category	B99	Non so/non pertinente	6	113	5.3	No
OA3	Rationale	C1	Qualità e sicurezza delle cure	17	114	14.9	No
OA3	Rationale	C2	Cura centrata sul paziente e presa in carico globale	22	114	19.3	No
OA3	Rationale	C3	Comunicazione efficace e linguaggio comune	8	114	7.0	No

Question	Axis	Code	Category	n	Denominator	Percentage	Inductively added category
OA3	Rationale	C4	Conoscenza e rispetto di ruoli/competenze	13	114	11.4	No
OA3	Rationale	C5	Preparazione al lavoro in team e al mondo del lavoro	35	114	30.7	No
OA3	Rationale	C6	Crescita professionale e personale	18	114	15.8	No
OA3	Rationale	C7	Efficienza organizzativa e uso delle risorse	6	114	5.3	No
OA3	Rationale	C8	Clima relazionale e benessere	4	114	3.5	No
OA3	Rationale	C9	Sviluppo del sistema sanitario e accademico	8	114	7.0	No
OA3	Rationale	C10	Riserve, condizioni e rischi	4	114	3.5	No
OA3	Rationale	C11	Gestione della complessità e ampiezza integrativa	11	114	9.6	Yes
OA3	Rationale	C99	Nessun razionale espresso	17	114	14.9	No
OA3	Intensity	I4	Maximum/indispensable	21	114	18.4	No
OA3	Intensity	I3	Very important	47	114	41.2	No
OA3	Intensity	I2	Important	15	114	13.2	No
OA3	Intensity	I1	Conditional importance/with reservations	5	114	4.4	No
OA3	Intensity	I0	No explicit intensity judgement	26	114	22.8	No

Note. Counts describe coded category salience within the available voluntary-response corpus and are not population prevalence estimates. OA3 intensity and rationale were coded on separate axes.

Figure S1. Flow of participants into the descriptive, measurement, regression, and nested analyses.

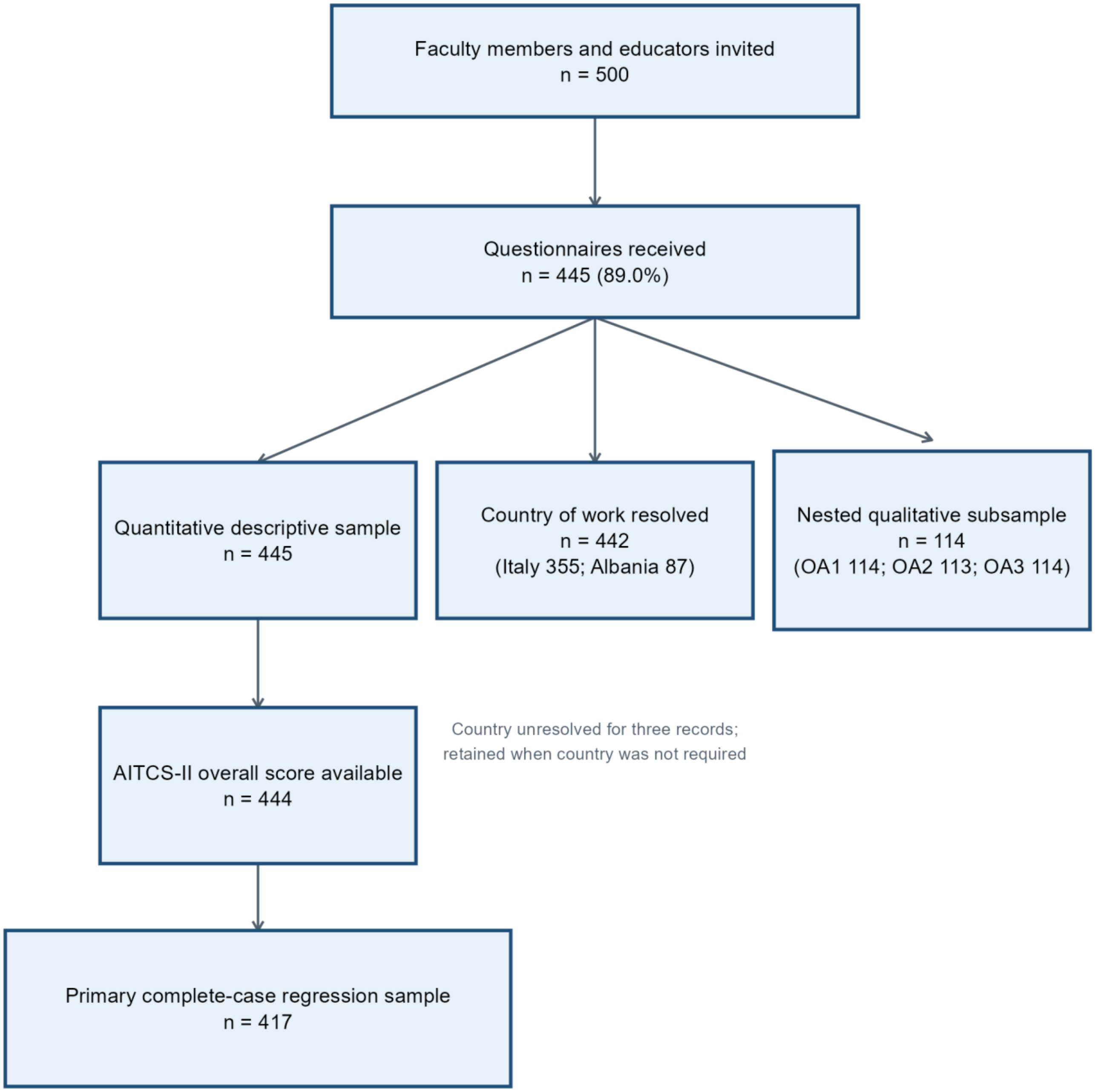

Note:
Invited: n = 500
Questionnaires returned:
n = 445 No questionnaire
returned: n = 55
Reasons for non-participation not collected

Figure S2. Variable-level missing-data profile.

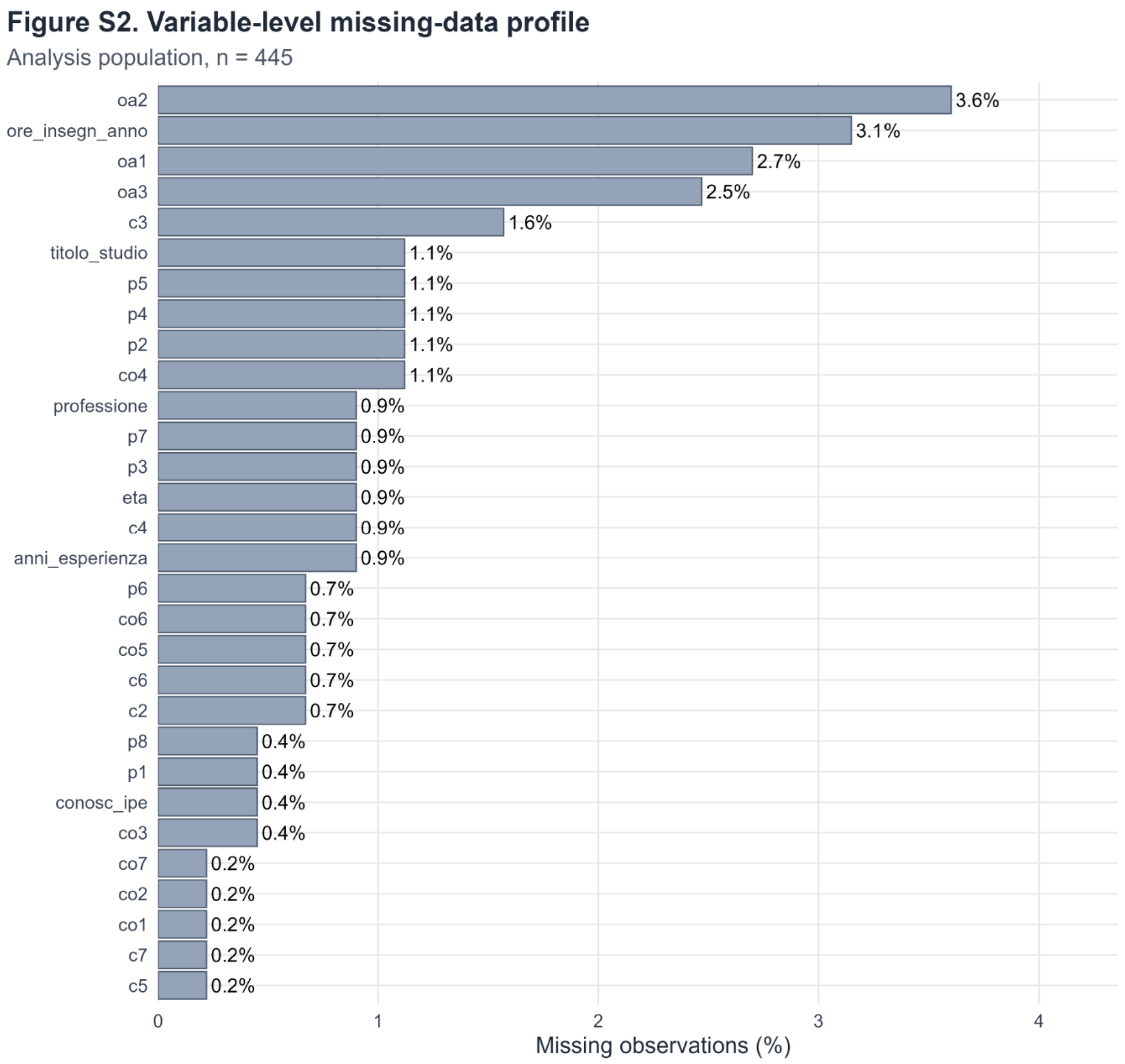

Figure S3. Standardised factor loadings for the correlated three-factor AITCS-II model.

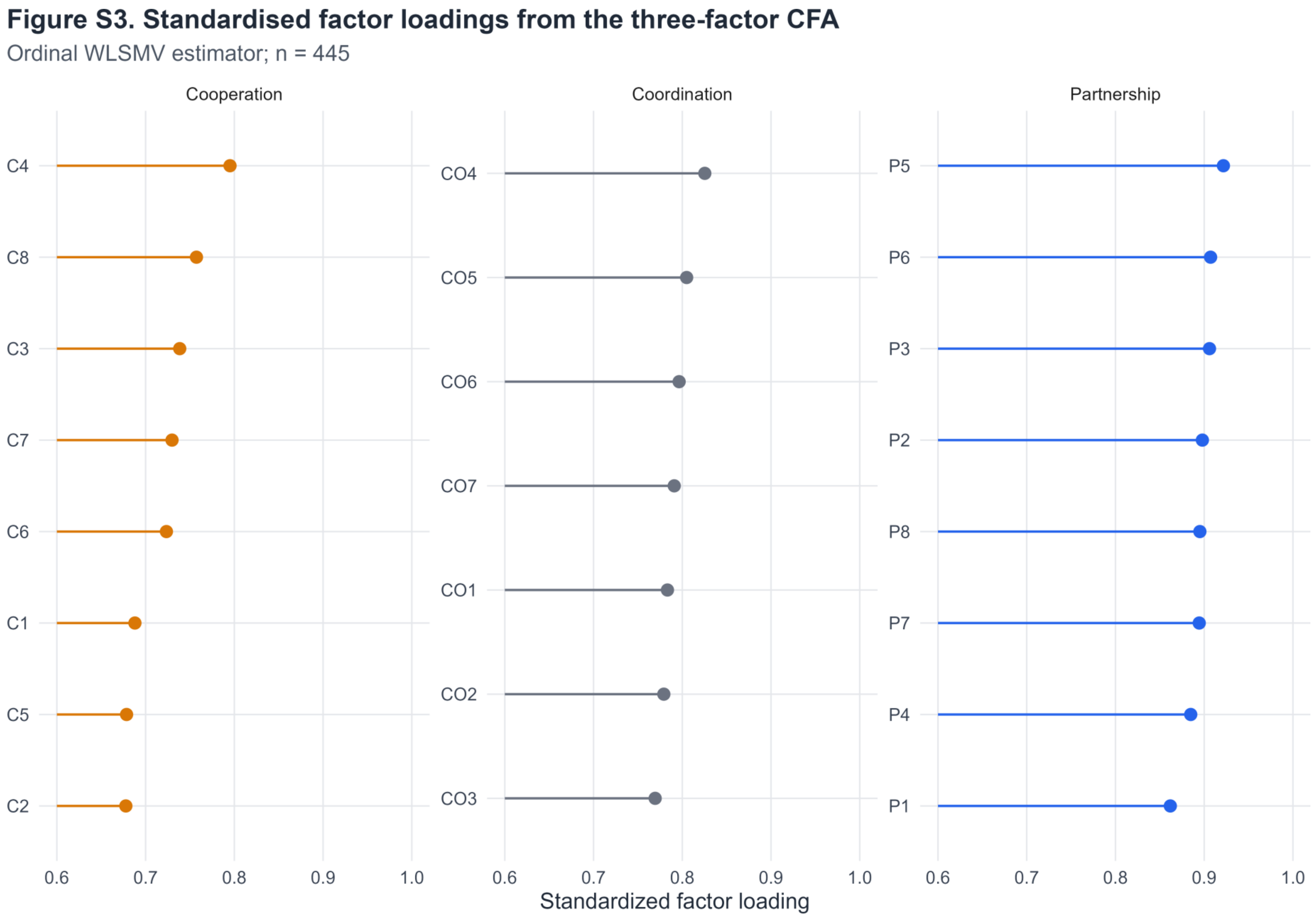

Figure S4. Spearman correlation heatmap for the main continuous variables.

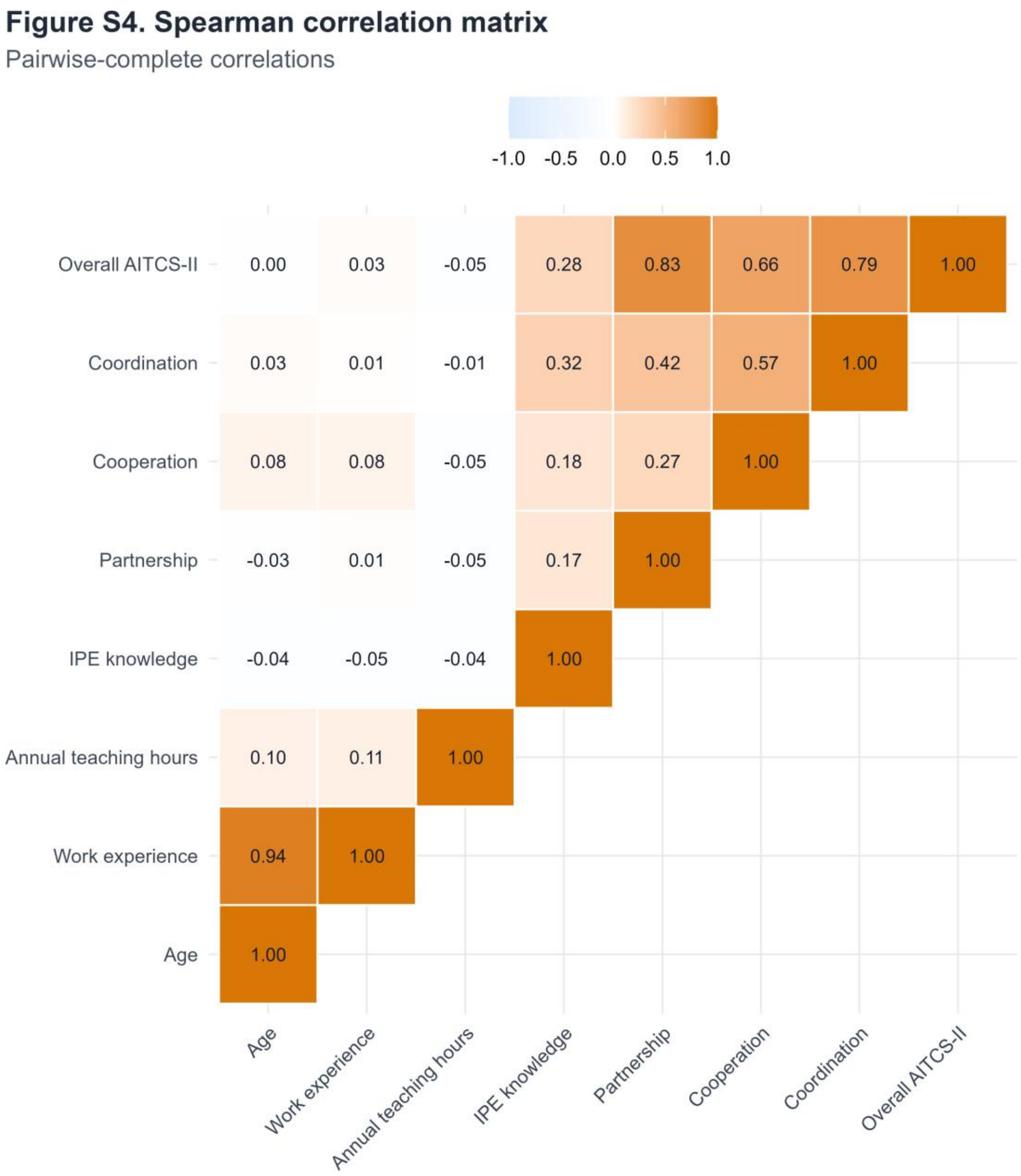

Figure S5. Diagnostic plots for the final AITCS-II interaction model.

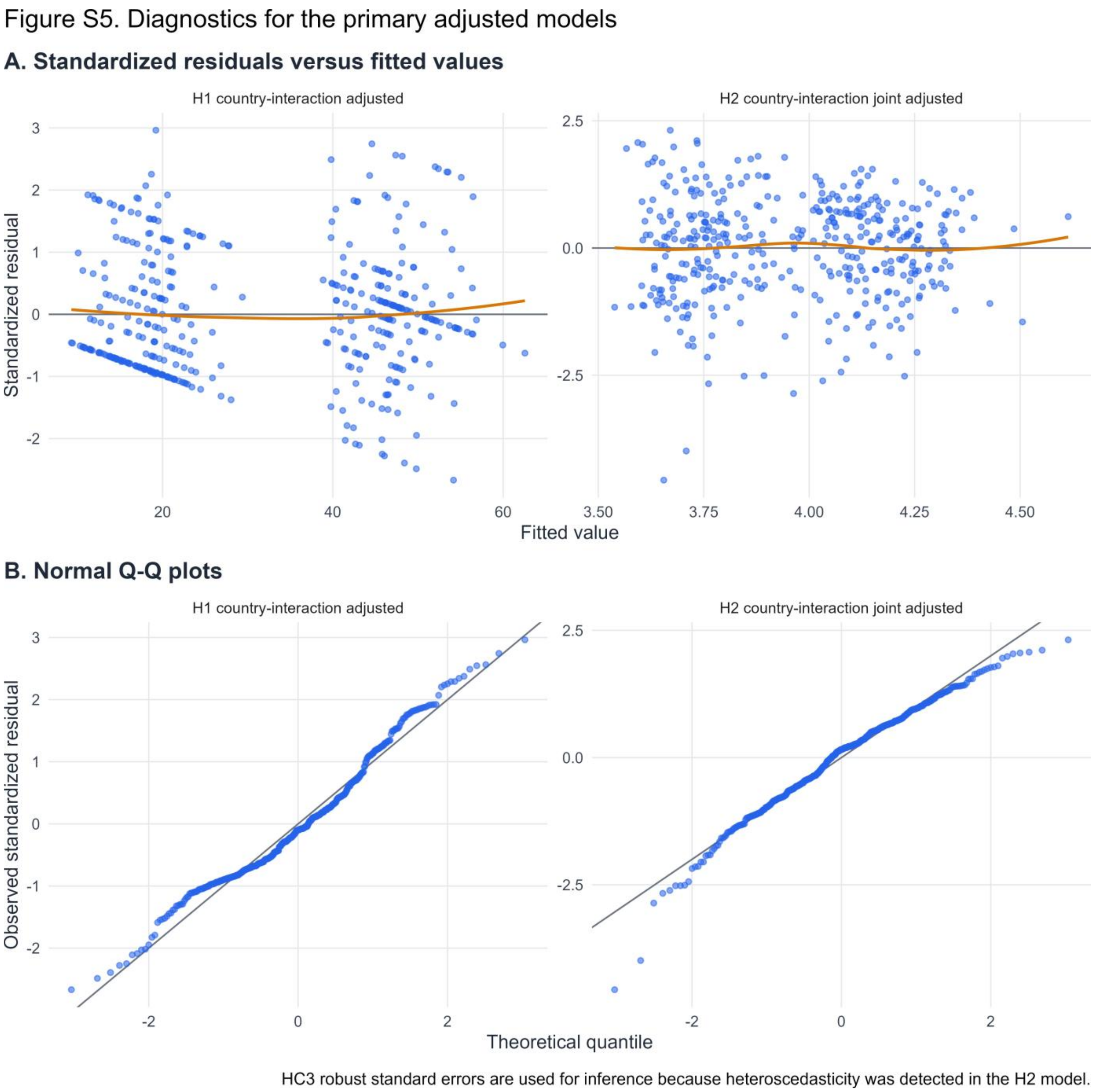

Supplement: Supplementary file 1 [file healthcare-14-02649-s001.zip › Supplementary Tables and Figures.pdf]
